# Integration of Phenomics and Metabolomics Datasets Reveals Different Mode of Action of Biostimulants Based on Protein Hydrolysates in *Lactuca sativa* L. and *Solanum lycopersicum* L. Under Salinity

**DOI:** 10.3389/fpls.2021.808711

**Published:** 2022-02-03

**Authors:** Mirella Sorrentino, Klára Panzarová, Ioannis Spyroglou, Lukáš Spíchal, Valentina Buffagni, Paola Ganugi, Youssef Rouphael, Giuseppe Colla, Luigi Lucini, Nuria De Diego

**Affiliations:** ^1^Photon Systems Instruments (PSI), spol. S.r.o., Drásov, Czechia; ^2^Department of Agricultural Sciences, University of Naples Federico II, Portici, Italy; ^3^Plant Sciences Core Facility, Central European Institute of Technology, Masaryk University, Brno, Czechia; ^4^Centre of the Region Haná for Biotechnological and Agricultural Research, Czech Advanced Technology and Research Institute, Palacký University, Olomouc, Czechia; ^5^Department for Sustainable Food Process, DiSTAS, Università Cattolica del Sacro Cuore, Piacenza, Italy; ^6^Department of Agriculture and Forest Sciences, University of Tuscia, Viterbo, Italy

**Keywords:** vegetal-based protein hydrolysates, multivariate statistical analysis, metabolomics, secondary metabolism, salt stress, *Lactuca sativa* L., *Solanum lycopersicum* L., high-throughput phenotyping

## Abstract

Plant phenomics is becoming a common tool employed to characterize the mode of action of biostimulants. A combination of this technique with other omics such as metabolomics can offer a deeper understanding of a biostimulant effect *in planta*. However, the most challenging part then is the data analysis and the interpretation of the omics datasets. In this work, we present an example of how different tools, based on multivariate statistical analysis, can help to simplify the omics data and extract the relevant information. We demonstrate this by studying the effect of protein hydrolysate (PH)-based biostimulants derived from different natural sources in lettuce and tomato plants grown in controlled conditions and under salinity. The biostimulants induced different phenotypic and metabolomic responses in both crops. In general, they improved growth and photosynthesis performance under control and salt stress conditions, with better performance in lettuce. To identify the most significant traits for each treatment, a random forest classifier was used. Using this approach, we found out that, in lettuce, biomass-related parameters were the most relevant traits to evaluate the biostimulant mode of action, with a better response mainly connected to plant hormone regulation. However, in tomatoes, the relevant traits were related to chlorophyll fluorescence parameters in combination with certain antistress metabolites that benefit the electron transport chain, such as 4-hydroxycoumarin and vitamin K1 (phylloquinone). Altogether, we show that to go further in the understanding of the use of biostimulants as plant growth promotors and/or stress alleviators, it is highly beneficial to integrate more advanced statistical tools to deal with the huge datasets obtained from the -omics to extract the relevant information.

## Introduction

Changes in climate patterns are dramatically influencing some agricultural areas with special impact in arid, semi-arid, and coastal agricultural areas (Corwin, 2020). Soil salinity already covers 20% of total cultivated and 33% of the irrigated agricultural lands worldwide, and is expected to increase at a faster rate than now by the year 2050 (Mukhopadhyay et al., 2021). The high salt concentration in the soil reduces plant growth and, hence, yield in two ways: increasing the osmotic potential of the soil solution, making it harder for the plant to extract water, and accumulating into the root and shoot tissue at a concentration that can be toxic for the plant (Munns and Tester, 2008). The extent of salinity damage to the fitness and final yield of the crop can change according to the species. For example, lettuce (*Lactuca sativa* L.) reduces plant growth and yield under salt stress conditions (Moncada et al., 2020). However, tomatoes (*Solanum lycopersicum* L.) can maintain the fruit yield and increase their quality under moderate stress (Meza et al., 2020), whereas severe salt stress reduced tomato growth and provoked severe damages, especially in young seedlings (Ali et al., 2021).

To reduce the yield loss connected to salinity, scientists are moving toward the selection of more tolerant genotypes through breeding, genetic engineering, and marker-assisted selection (MAS) (Munns and James, 2000; Yamaguchi and Blumwald, 2005). However, these methods are expensive, time-consuming, and, in the case of genetic engineering, received with suspicion by the general public (Yamaguchi and Blumwald, 2005; Halford and Shewry, 2000). A more sustainable alternative is represented by the use of protein hydrolysates (PHs), a class of non-microbial plant biostimulants obtained from the partial hydrolysis of protein sources of plant or animal origin (Colla and Rouphael, 2015). Many works from the last years have enlightened the effects of PHs as stress alleviators on different crops growing in saline conditions (Van Oosten et al., 2017; Dell’Aversana et al., 2020; Di Mola et al., 2021). Nonetheless, it is important to remember that the effects of the PHs on crops can vary with the plant species or varieties, the time of the application, and the dose (Lisiecka et al., 2011).

Before a new potential PH-based biostimulant is put on the market, it is essential to test its effects in multiple conditions and on different crops. High-throughput automated platforms for plant phenotyping have proven to improve and speed up the biostimulant testing process (Rouphael et al., 2018). Different sensors can be implemented in high-throughput phenotyping platforms, allowing the user to monitor the effects of the PH applications on many morpho-physiological traits throughout the entire crop life cycle (Paul et al., 2019a,b). As a result, we can define in which crop, developmental stage, and dosage the application is recommended. Besides, a deeper physiological study allows the characterisation of their mode of action. This information can be used for further possible applications.

The use of other -omic approaches, such as metabolomics, allows studying the biostimulant effect in a more complex manner, supporting, and integrating the phenomics data to better understand the biochemical processes activated in the plants by the biostimulants application. However, the data analysis and interpretation of the complex omics datasets can become another challenging bottleneck. Here, we investigated the mechanism of action of a set of 7 PHs in lettuce and tomato subjected to early and late salinity stress. We hypothesise that salinity will reduce plant growth and change the physiology of the plant in tomato and lettuce. However, the PH application will ameliorate the salt negative effect in both plant species. Besides, a deep data analysis using advanced statistical tools will allow us (i) to understand better the effect of the PHs on two species, lettuce and tomato, selected for their economic importance, their distinct architecture, and purpose, and their different sensitivity to salinity stress, (ii) to evaluate the biological translation from the results obtained in PH-primed Arabidopsis grown under salt stress (Sorrentino et al., 2021) to other crops under similar growing conditions, and (iii) to demonstrate the necessity of the use of statistical approaches to simplify complex omic datasets allowing identification of the traits relevant for the understanding of a biostimulant mechanism of action.

## Materials and Methods

### Plant Material and Growing Conditions

Seeds of *Lactuca sativa* L. var. capitata (Salanova cv Aquino) and *Solanum lycopersicum* L. cv MicroTom were sown in 250 ml pots filled with 235 g of a mixture of sieved peat (Substrate 2, Klasmann-Deilmann GmbH, Geeste, Germany) and river sand in 1:1 proportion. All the pots were watered up to 55% of the soil relative water content (SRWC). The water holding capacity of the substrate was calculated as described by Junker et al. (2015). The covered pots were stratified at 4°C in the dark for two days. After that, the pots were moved to a climate-controlled growth chamber (FS-WI, Photon Systems Instruments, Czechia) under long-day conditions (16 h light/8 h dark). The climate conditions in the growth chamber were set at 21/19°C for day/night temperature with 60% relative humidity (RH) and 120 μmol m^–2^ s^–1^ cool-white LED (6,500 K) and 5.5 μmol m^–2^ s^–1^ far-red LED (735 nm) lighting. These conditions were kept constant throughout the entire experiment. The pots were kept covered with a plastic lid for the first 24 h to maintain the soil moisture before it was removed.

### Selection of the Plants

Eight days after lettuce stratification and ten days after tomato stratification, when most of the germinated seedlings had reached the 2-true-leaf stage, a top view RGB picture of all plantlets was taken using the top view RGB2 camera in the PlantScreen™ Compact system (Photon Systems Instruments, Brno, Czechia). The plants with areas between the 1st and the 4th quartile of the normal distribution of the population were used for the experiment. In the tomato experiment, each variant counted 6 plantlets as biological replicates, with a total of 96 plants. For the lettuce experiment, each variant counted 8 plantlets as biological replicates, with a total of 128 plants.

### High-Throughput Phenotyping

**FIGURE 1:**
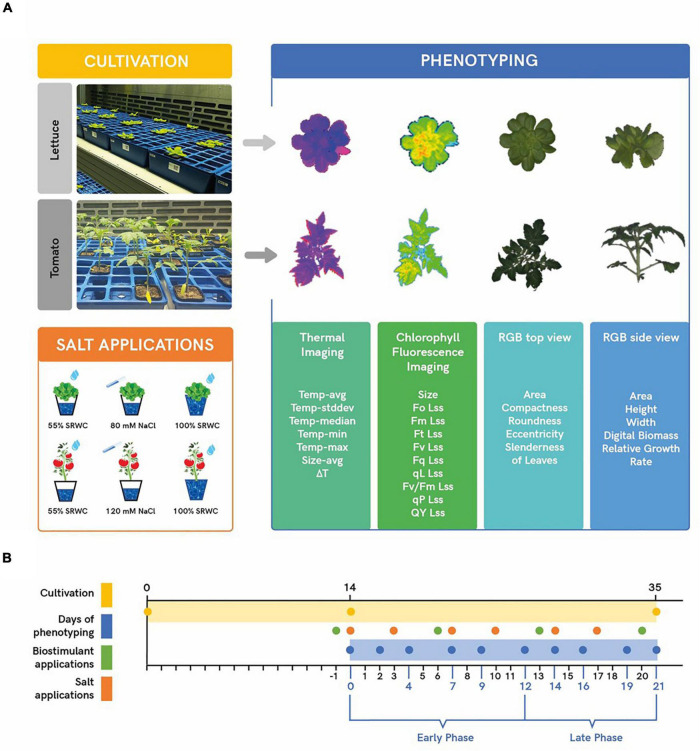
Scheme of plant cultivation and phenotyping protocol. Plants were manually transferred from the cultivation chamber to PlantScreen™ Compact System for imaging using four different sensors (thermal camera, chlorophyll fluorescence, and top and side RGB). The resulting false-color, segmented images, and list of extracted parameters obtained from the sensors are shown **(A)**. Timeline of plant cultivation (yellow bar) and phenotyping protocol (blue bar). Green dots show four-time points of the foliar application for the selected biostimulants and the orange dots show six timepoints for the salt treatment **(B)**.

To investigate the effects of PHs application on the morpho-physiological parameters of lettuce and tomato grown under salt stress conditions, trays containing two pots with one plantlet each were automatically transported within PlantScreen™ Compact System on conveyor belts between the light-isolated imaging cabinets, weighing and watering station, and the dark/light acclimation chamber. The trays were measured thrice a week, ending with 10 phenotyping rounds distributed in 21 days for lettuce and 24 days for tomato (Figure 1), with the starting point before the first salt application (Day of Phenotyping 1, henceforth defined as DoP 1). The phenotyping protocol used was the same for both crops. Physiological measurements [Chlorophyll Fluorescence (ChlF) and Thermal Imaging (IR)], being sensitive to circadian rhythm regulation mechanisms (Cano-Ramirez and Dodd, 2018), were always performed in the morning. A single round measuring protocol consisted of an initial 15 min light-adaptation period inside the acclimation chamber, followed by IR, and red-green-blue (RGB) top view imaging (RGB2). Next, 15 min dark-adaptation was applied, followed by chlorophyll fluorescence kinetic imaging, RGB side view imaging (RGB1), and weighing and watering (Figure 1A). Due to the limited capacity of the phenotyping system for the lettuce experiment, the trays were divided into 3 blocks with 16 trays each. The measuring round for one block lasted for 2 h and 45 min. The PlantScreen™ Analyser software (PSI, Czechia) was used to automatically process, re-analyse, and export the data.

### Biostimulants Selection and Application

Seven PHs obtained by enzymatic hydrolysis of vegetal-derived proteins were selected from a batch of eleven PHs that were previously screened for their mode of action (Sorrentino et al., 2021) and used for the experiment. They included PHs from different plant sources belonging to the botanical families of Fabaceae (O), Malvaceae (C), Brassicaceae (F), Solanaceae (B), and Graminaceae (P), and two commercial products [Trainer^®^ (D) and Vegamin^®^ (H)], commercialised by Hello Nature Inc. (former Italpollina) (Anderson, IN, United States) and used as positive controls. The PH was obtained through enzymatic hydrolysis of the dry biomass and was then analysed for their total nitrogen and carbon content. For a detailed description of the procedure, see Sorrentino et al. (2021) and Ceccarelli et al. (2021).

Biostimulants were applied to leaves through spraying once a week, using only distilled water for the controls or the given PH in a concentration of 3 mL/L for the treated plants. A total of 4 foliar applications of PHs were done throughout the experiment (Figure 1B). The first spraying (priming) was performed one day before the first salt application. Two hours before and after the spraying of the plants, the relative humidity in the growing chamber (FS-WI) was increased up to 80% to promote the stomata opening.

Due to the limited capacity of the phenotyping platform, the lettuce experiment was divided into two rounds, each consisting of 64 plants. The substances B, C, and F were tested in the first round, while D, H, O, and P in the second round.

### Watering and Salt Treatment

All the pots were watered after each phenotyping round at up to 55% SRWC with a modified Hoagland solution [0.36 g/L Ca (NO_3_)_2_, 0.1 g/L KH_2_PO_4_, 0.80 g/L KNO_3_, 0.04 g/L NH_4_NO_3_, 0.13 g/L MgSO_4_, and 0.01 mg/L of MIKROM fertilizer (Cifo Srl, S.Giorgio di Piano (BO), Italy)] using the Weighing and Watering station in the PlantScreen™ Compact. The solution was freshly prepared before each watering round and the pH was adjusted to a value of 5.7.

Starting from 2 weeks after stratification, the plants belonging to the stress group were subjected twice a week to salt application, ending with 6 applications for both crops (Figure 1B). In the lettuce assay, our objective was to reach a concentration of 40 mM NaCl in the soil, corresponding to moderate stress (Freitas et al., 2019). To avoid osmotic shock to the plants and NaCl accumulation in the soil, all the pots were first watered up to 55% of SRWC with plain nutrient solution. The plants belonging to the stress group were then given 40 ml each of an 80 mM NaCl solution (8.7 mS/cm). In the end, after a couple of hours from the salt application, all the plants were watered again of up to 100% of their SRWC to create drainage of the solution from the pot. The same setup was used with tomato, but in this case, the salt solution increased to 120 mM NaCl (14 mS/cm) to reach a concentration of around 60 mM NaCl in the pot, corresponding to moderate salt stress (Meza et al., 2020; Figure 1B). The two NaCl concentrations used in the experiments were the result of several preliminary tests conducted on both crops (data not shown).

### Imaging Protocol and Data Analysis

#### Red-Green-Blue Imaging

Red green blue imaging using high-resolution top-view and side-view RGB cameras and an optimised image segmentation algorithm for automated analysis were used to calculate the number of plant-specific pixels throughout the whole experiment. The RGB images were processed as described by Awlia et al. (2016) and Paul et al. (2019a,b).

Projected shoot area (PSA) from the top (PSA_top_) and the side view (PSA_side_) was used to calculate the Digital Biomass (DM) of each plant (Rahaman et al., 2017):


$$\sqrt{P\,S\,A_{s\,i\,d\,e}^{2}\times P\,S\,A_{t\,o\,p}}$$

Digital Biomass, corresponding to the approximate volume, was then used to calculate the Relative Growth Rate (RGR), where*T*_1_ and *T*_*2*_ indicate the time interval (days), while *DM*_1_ and *DM*_2_ indicate the corresponding digital biomass:


$$\left(\ln D\,M_{2}-\text{ln}\,D\,M_{1}\right)/\left(T_{2}-T_{1}\right)$$

Relative growth rate (RGR) was calculated twice during the experiment: from DoP 0 to DoP 12 (**Early Phase**) for both crops, and from DoP 12 to DoP 21 for lettuce plants, or to DoP 24 for tomato plants (**Late Phase**).

#### Chlorophyll Fluorescence Imaging

To assess the effects of salt stress and biostimulants application on the photosynthetic performance of the plants, ChlF measurements were acquired using an enhanced version of the FluorCam FC-800MF pulse amplitude modulated (PAM) chlorophyll fluorometer incorporated into the PlantScreen™ Compact System (for more details, see Henley, 1993). After 15 min of dark adaptation, the light curve protocol, as described in Awlia et al. (2016), was used to quantify the rate of photosynthesis at different photon irradiances (Rascher et al., 2000). Four actinic light irradiances [Lss (Light steady-state) 1: 180 μmol m^–2^ s^–1^; Lss2: 480 μmol m^–2^ s^–1^; Lss3; 780 μmol m^–2^ s^–1^ and Lss4: 1,080 μmol m^–2^ s^–1^] with a duration of 60 s were used to quantify the rate of photosynthesis. The raw data were automatically processed using the PlantScreen™ Analyser software (PSI, Brno, Czechia). From the measured fluorescence transient states, the basic ChlF parameters were derived (i.e., F0, Fm, Ft, and Fv), which were used to calculate a range of parameters characterizing the plant photosynthetic performance (i.e., Fv/Fm, Fv′/Fm′, NPQ, and ΦPSII). We chose to evaluate the parameters obtained after the exposure of the plants to the light of intensity 480 μmol m^–2^ s^–1^ (Lss2) since they provide the highest discriminative power between control and stress plants.

#### Thermal Imaging

To determine the leaf temperature of the plants, we used the thermal imaging unit implemented into the PlantScreen™ Compact system, which allows assessing the canopy temperature from the top view. The thermal imaging unit incorporated in the PlantScreen™ Compact System consists of a light-isolated box with one top view camera mounted on a static frame and a temperature sensor to increase contrast for the image-processing step. The imaged area is 440 mm × 340 mm (height × width). To assess the Spatio-temporal variations in temperature over plant surface, we used an FLIR A615 thermal camera with 45° lenses and a resolution of 640 × 480 pixels and high-speed infrared windowing option and <50 mK thermal sensitivity (FLIR Systems Inc., Boston, MA, United States). The canopy temperature of each plant was automatically extracted with PlantScreen™ Analyser software (PSI, Brno, Czechia) by mask application, background subtraction, and pixel-by-pixel integration of values across the entire plant surface area.

To minimize the influence of the differences in environmental conditions and image acquisition timing among individual plants, the average canopy temperature of each plant (T_avg_) was normalised with the actual temperature inside the Thermal Imaging box and expressed as canopy temperature depression or δT (°C) (Hou et al., 2019).

### Untargeted Metabolomic Analysis

At the end of the experiment, at DoP 21 and 24 in lettuce and tomato, respectively, the third true leaf of each plant was harvested and freeze-dried. The material from control plants and plants treated with the 7 PHs was used for the metabolomic analysis. Lyophilised plant material (50 mg for lettuce and 250 mg for tomato) was extracted in twenty volumes (w/v) of methanol/water solution (70:30, v/v), acidified with 0.1% formic acid by Ultra-Turrax (Polytron PT, City, Switzerland), centrifuged, and then filtered through a 0.22 μm membrane as previously reported (Paul et al., 2019a,b). Untargeted metabolomics was performed using a 6,550 iFunnel quadrupole-time-of-flight mass spectrometer and a 1,200 series ultra-high-pressure liquid chromatographic system (UHPLC-ESI/QTOF-MS) from Agilent Technologies (Santa Clara, CA, United States) as previously described (Miras-Moreno et al., 2021). Briefly, 6 μL were injected and a reverse-phase chromatography was applied under a water-acetonitrile gradient elution (6 to 94% acetonitrile in 33 min). The mass spectrometer worked in positive ionisation (ESI+) and in SCAN mode for the acquisition of accurate masses ranging from 100 to 1,200 m/z. Four replicates were analysed for each treatment and samples were randomly sequenced. Quality Controls (QCs) were prepared by pooling all the extracts and were analysed throughout the chromatographic sequence using the same chromatographic conditions as samples but were acquired in data-dependent MS/MS mode (1Hz, 50–1,200m/z, 12 precursors per cycle) at different collision energies (10, 20, and 40eV).

Agilent Profinder B.07 (Agilent Technologies) software was used for mass (5-ppm tolerance), retention time (0.05min maximum shift) alignment, and for processing all the mass features from UHPLC-ESI/QTOF-MS raw data. The combination of monoisotopic mass, isotopes accurate spacing, and isotope ratio was used for annotation using the PlantCyc 12.6 database (Plant Metabolic Network)^1^, as previously reported (Pretali et al., 2016; Schläpfer et al., 2017). Only those compounds identified in 75% of the replications within at least one treatment were retained. Thereafter, MS/MS confirmations from QCs were carried out using the software MS-DIAL 4.24 (Tsugawa et al., 2015), formerly using MS/MS experimental spectra available in the software (Mass Bank of North America), and then using MS-Finder *in silico* fragmentation (Tsugawa et al., 2016). The annotation process corresponded to level 2 of confidence as set out in the COSMOS Metabolomics Standards Initiative (Salek et al., 2015).

### Statistical Analysis

For the phenotyping data, statistical differences between treatments and time points were determined by Mixed model analysis (McCulloch and Searle, 2000; Boisgontier and Cheval, 2016) and multiple pairwise comparisons using *post hoc* Tukey’s test (*P*-value <0.05). The statistical analysis was implemented in R studio (R GUI 4.0.3) using the “lmer” and “emmeans” packages (R Core Team, 2014; Bates et al., 2015; Russell, 2020). Then, to define the specificities of each crop and their response to the foliar application with PHs, and to go further in the mode of action, hierarchical clustering was applied with the use of “Ward’s” linkage method to find similarities between crops, growth conditions, and the best and worse performed biostimulant and to identify clusters (Saxena et al., 2017). Finally, random forest classification was also applied to identify the significant variables for the treatment classification (Qi, 2012).

Concerning metabolomics, the software Agilent Mass Profiler Professional B.12.06 (from Agilent Technologies, Santa Clara, CA, United States) was used for data normalisation and baselining (Mimmo et al., 2017). Then, unsupervised hierarchical cluster analysis (HCA) based on fold-change heatmaps (Squared Euclidean distance) was used to naively describe patterns across treatments. Thereafter, supervised multivariate statistics were performed in SIMCA 13 (Umetrics, Malmo, Sweden), where orthogonal projection to latent structures discriminant analysis (OPLS-DA) was carried out. Each supervised model (separate models for tomato and lettuce, and then a comprehensive model for salt-stressed versus control plants), was validated by CV-ANOVA, checked for overfitting by permutation testing (*N* = 200), and then inspected for goodness-of-fit (R2Y) and prediction ability (Q2Y). After that, variable importance in projection (VIP) ranking was used to identify the most discriminant compounds in each OPLS-DA model. Finally, ANOVA (*P*-value <0.01, Bonferroni multiple testing correction) and fold-change (FC) analysis (FC ≥ 1.3) were combined into Volcano Plots, and differential compounds were imported into the Omic Viewer Pathway Tool of PlantCyc (Stanford, CA, United States) software (Caspi et al., 2013) for biochemical interpretations.

## Results

### Development of the Experimental Protocol for Salt and Biostimulants Applications

To effectively characterise the outcome of biostimulants applications on lettuce and tomato performance in the early vegetative growth phase, we first optimised the experimental protocol for plant cultivation, mild-salt stress application, and the stress response quantification. The crops were analysed in independent experiments as they are very diverse in their tolerance to salinity, and two different concentrations of NaCl in the nutrient solution were used to water the plants as described in Materials and Methods (Figure 1). Lettuce and tomato plants were grown for 35 and 39 days, respectively, and this period corresponded to the complete head maturation in lettuce and the beginning of the flowering stage in tomatoes.

**FIGURE 2:**
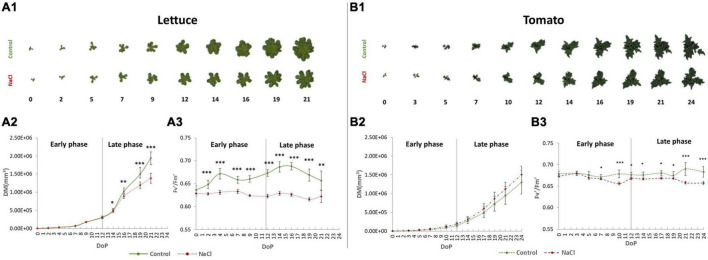
Salinity response in tomato and lettuce plants. The RGB top view colour-segmented images of lettuce **(A1)** and tomato **(B1)** control and salinity-stressed plants over the time-course of the experiment. Digital biomass (DB) of the lettuce **(A2)** and tomato **(B2)** plants. The maximum quantum efficiency of photosystem II in the light-adapted state (Fl′/Fm′) for lettuce [full line, **(A3)**] and tomato [dashed line, **(B3)**] plants. Values represent the average of 8 (lettuce) and 6 (tomato) biological replicates per treatment, error bars represent standard deviation. The significant differences between control and salt treatment are indicated with *, ^**^, and ^***^ for *p*-values below 0.05, 0.01, and 0.001, respectively. Data and images shown are from the 1st round of the lettuce experiment.

The PHs were applied *via* foliar spraying with solutions of 3 mL/L each (Figure 1; Di Mola et al., 2019a,b). The morpho-physiological traits of the plants were quantified dynamically throughout the trial to monitor their growth performance and physiological status. As a result, we could clearly distinguish two periods in the experiment, an early and late phase, in which the response of the plants to the salt stress and to the interaction with the biostimulants applications were diverse. It is well-known that plants respond to salt stress in two phases (Ugena et al., 2018): a rapid and osmotic phase, described here as the early phase (DoP 0-12), and a slower ionic phase due to the ion toxicity, referred to as the late phase (DoP 12-21 in lettuce or DoP 12-24 in tomato) (Figure 2).

### Lettuce and Tomato Plants Have Different Physiological Responses to Mild Salinity

In lettuce, salt stress reduced plant growth in the later phase of the experiment after 4 salt applications (after DoP 14) (Figures 2A1,A2), and the reduction was similar in the lettuce plants from the first and second trials with 35 and 29% lower digital biomass (DB) than their correspondent controls, respectively (Supplementary Figures 1A1,B1). We further assessed other morphological parameters, such as roundness, compactness, and slenderness of the leaves (SOL), showing that they did not differ between the rounds, but differed between the controls and salt-stressed plants in the late phase, with the less compact, round, and more SOL in the stressed lettuce (Figure 2A1 and Supplementary Figures 1A,B).

The photosynthetic performance of the plants during the development and with the progression of the salt stress was also affected (Figure 2A3 and Supplementary Figures 1C,D). In the two rounds that were analysed, the most significant differences were observed in the late phase between the controls and the NaCl-treated lettuce plants. We showed that the maximum quantum yield of PSII photochemistry for the light-adapted (Fv′/Fm′) state and PSII operating efficiency (ΦPSII) were significantly reduced in the stressed plants, whereas the non-photochemical quenching (NPQ) was increased compared to the controls (Figure 2A3 and Supplementary Figures 1C,D). Altogether, our data demonstrate that in lettuce, only the late phase of salt stress imposition (after DoP12) was important to detect the differences in growth and in fluorescence-related parameters between treatments.

In tomato plants, the growth of the plant was not affected by the mild salt stress (Figures 2B1,B2). The remaining morphological parameters did not show differences between control and salt stress, except for the higher slenderness of the leaves (SOL) in salt-stressed plants during the transition from the early to late phase (Supplementary Figures 2A1–A4). Regarding the physiology, however, there were significant differences in several fluorescence-related parameters at the end of the early phase and late phase of the salt stress (Figure 2B3 and Supplementary Figure 2B). In salt-stressed tomatoes, we observed a significant reduction in Fv′/Fm′ and ΦPSII, and increased NPQ values compared to the controls.

### Protein Hydrolysates Specifically Improve Growth Performance of Lettuce Plants

**FIGURE 3:**
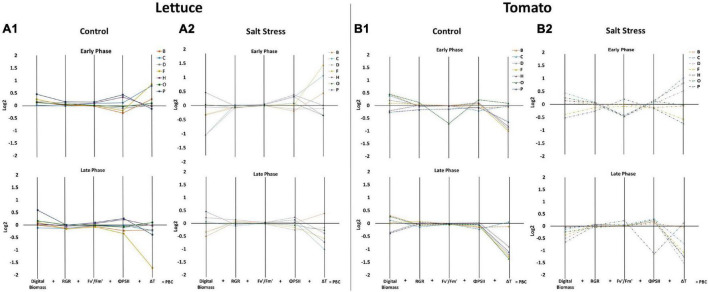
Characterisation of the effect of biostimulants on the performance of tomato and lettuce plants grown under control and salinity conditions. Parallel coordinate plots of the 5 main morpho-physiological parameters (Digital Biomass, RGR, Fv′/Fm′, ΦPSII and △T) are shown for lettuce plants grown in control [full lines, **(A1)**] and salt stress conditions [dotted lines, **(A2)**], for tomato plants grown in control [dashed lines, **(B1)**], and salt stress conditions [dotted and dashed lines, **(B2)**]. The values represent the log2 of the ratio between the plants treated with the 7 protein hydrolysates (PHs) and their respective controls, the sum of the resulting 5 values corresponds to the Plant Biostimulant Characterisation (PBC) index, used for the characterisation of the PHs. Data are shown for the early (0–12 Days of Phenotyping) and the late phase (12–21 Days of Phenotyping) of the plant growth.

In the following step, we analysed how the foliar application of selected PHs could modify the responses observed in salt-stressed and non-stressed plants. In lettuce plants, the substance P increased the DB at the late phase of stress after 3 foliar applications under both growth conditions (DoP13) (Figures 3A1,A2, Supplementary Figure 3B1, and Supplementary Tables 1A,B). The substances D and H also improved the DB, but only when plants were grown under salt stress conditions. Other morphological traits, such as roundness, compactness, or SOL, did not change (Supplementary Figures 3A2–A4,B1–B4 and Supplementary Tables 1A,B). Similarly, the foliar application with substance P increased the RGR of the plants in the early phase under control conditions (Figures 3A1,A2 and Supplementary Figures 6A1,A2). Other PHs did not modify the RGR of the plants compared to their respective controls (salt or control) in both the early and late phases. Interestingly, application of the PHs increased the final biomass (Supplementary Figures 6B,C), especially the dry weight of the aerial part of the plants when the substances B, C, F, and P were applied to plants grown under control and salt stress conditions, or when the substance O was applied under control conditions.

The application of biostimulants had a mild impact on the photosynthetic performance of the lettuce plants both under control and salt stress conditions. We showed that the plants treated with the substances P and H increased Fv′/Fm′ and ΦPSII values both under control and stress conditions, while we observed reduced NPQ levels along with the experiment (Figures 3A1,A2, Supplementary Figures 3C,D, 4C,D, and Supplementary Table 2A).

In tomato plants, the application of PHs did not have any effect on the morphological traits; the differences in DB, RGR, and fresh and dry weight were only due to the growth conditions. Digital biomass (DB), along with the fresh weights of the plants, were similar in PH-treated plants and control plants during both phases of the experiment (Figures 3B1,B2, Supplementary Figures 5A,B, 7, and Supplementary Table 1C). Similarly, no significant improvement of the photosynthetic efficiency of the plants was observed in tomato plants sprayed with PHs in any growth conditions (Figures 3B1,B2, Supplementary Figures 5C,D, and Supplementary Table 2C).

We have further analysed the impact of PHs applications on the leaf surface temperature profile of both crops using thermal image analysis. The salt stress significantly increased the temperature of the leaf surface in lettuce but not in tomato plants (Supplementary Figure 8). Similarly, the changes of the temperature by the application with biostimulants were more visible in lettuce than in tomato plants. We showed that the lettuce plants treated with the substances C and F had a significantly reduced surface temperature when grown both under control and stress conditions (Figures 3A1,A2, Supplementary Figures 8A,B, and Supplementary Table 3A). In tomatoes, the biostimulants reduced the temperature of the leaf surface after the first application in plants that were under control and salt stress conditions. However, in the late phase, they increased the temperature over the respective control in almost all the cases (Supplementary Figure 8C and Supplementary Table 3A).

### Investigating the Mode of Action of the Protein Hydrolysates Through the Plant Biostimulant Characterisation Index

To simplify the results and to identify the specific mode of action of the 7 PHs, we used the Plant Biostimulant Characterisation (PBC) index as described previously (Ugena et al., 2018; Sorrentino et al., 2021). For the calculation of the PBC indexes, we selected the five traits (DB, RGR, Fv′/Fm′, ΦPSII, and ΔT) that provided the highest discriminative power between the different treatments (Supplementary Tables 1–3). The PBC indexes for the Early Phase (from DoP 0 to 12) and the Late Phase (from DoP 12 to 21 in lettuce or from DoP 12 to 24 in tomato) were calculated independently since we could observe different responses of the plants treated with the 7 PHs in the two phases. To determine the index value, the log2 of the ratio between the biostimulant treated plants and untreated ones was determined for each crop and growth conditions, and then represented in parallel plots (Figure 3). Then, the five obtained values represented in each parallel plot were summed to end with a unique number that represents the PBC index (for further detail see Sorrentino et al., 2021) which was included in Table 1. The calculated PBC index for each compound, growing condition, and phase of the trial could be negative (red) or positive (blue), providing information about the mode of action of that specific combination (Table 1). More in detail, the substances with positive PBC indexes (darker blue) in control conditions are characterised as plant growth promoters, whereas the negative (darker red) are plant growth inhibitors. Overall, our data clearly showed that in lettuce plants, the substance P was both the best growth promotor and stress alleviator, improving the fitness of the plants in all growing conditions and both stages of the trial. The second best was H, while the absolute worst was B (Table 1A). Some of the other PHs proved to be beneficial to the crop only in a specific growing condition and/or phase of the trial. For example, F showed an effect as a growth promotor, but only in the late phase of the experiment, while O acted as a stress alleviator in the early phase, but its effect fainted in the Later Phase (Table 1A).

**TABLE 1:** Classification of 7 protein hydrolysates (PHs) using Plant Biostimulant Characterisation (PBC) index.

	(A) Lettuce				(B) Tomato			
	*Control*		*NaCl*		*Control*		*NaCl*	
	Early phase	Late phase	Early phase	Late phase	Early phase	Late phase	Early phase	Late phase
*B*	−0.38	−0.21	−1.04	−0.78	0.35	0.18	0.09	0.10
*C*	−0.54	−0.07	−1.73	1.01	0.17	−0.32	0.84	1.00
*D*	0.12	−0.10	−1.18	−0.42	1.27	1.14	1.14	1.33
*F*	−0.73	1.23	−1.77	0.15	1.21	1.24	0.92	1.00
*H*	0.63	0.30	0.53	1.00	0.73	0.63	1.36	0.77
*O*	0.15	−0.02	0.38	0.19	1.96	1.45	1.49	1.23
*P*	1.37	1.32	0.84	1.29	0.60	0.64	0.34	0.36

*The PBC index values for each substance, in Early (0-12 Days of Phenotyping for both crops) and in Late Phase of the experiment (21-21 or 12-24 Days of Phenotyping for lettuce or tomato, respectively). The PBC index of the 7 studied PHs in lettuce (A) and tomato (B) plants under control and salinity conditions. White corresponds to 0, positive values are highlighted in blue and negative values are highlighted in red, the farther the value from 0, the darker is the corresponding hue.*

For tomato, the absolute best performer was the substance O, followed by D, F, and H, all acting as growth promotors and stress alleviators. Contrarily, the plants treated with the substance B showed the worst performances, especially in salt stress conditions (Table 1B).

**FIGURE 4:**
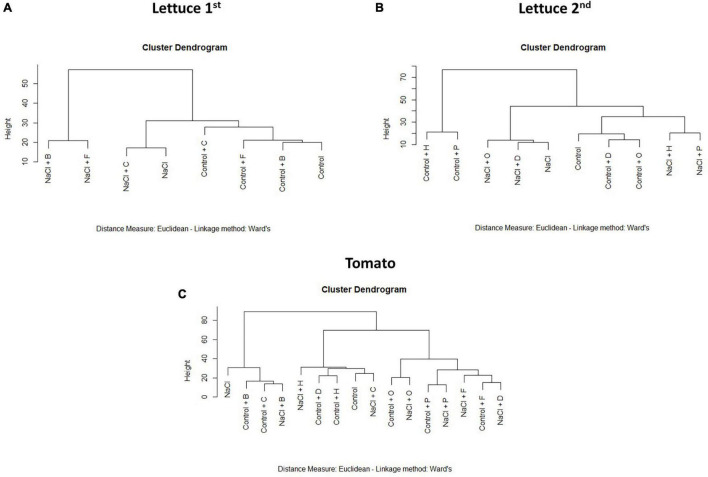
Cluster Dendrograms for all phenotypical data. Cluster analysis of the lettuce [**(A)** 1st round and **(B)** 2nd round] or tomato **(C)** plants treated with 7 different PHs and grown under control and salt stress conditions.

The results obtained from the PBC index were also corroborated by a cluster analysis performed with the complete phenotyping data set (Figure 4). In lettuce, the plants treated with H and P were located in an independent cluster that was divided into two subclusters due to the growth conditions (control or salt) but independent of the stress and unstressed control plants (Figure 4A). Contrarily, the rest of the substances were located with their respective controls that were only separated by the stress effect (Figures 4A,B). In tomato plants, except for the substances B and C, all PHs were beneficial for the plant fitness, especially when they were grown under salt stress conditions (Figure 4C). Altogether, we could conclude that H and P were the best growth promotors and stress alleviators, whereas B was a plant growth inhibitor.

### The Applications of Protein Hydrolysates Activate Different Metabolic Pathways in Lettuce and Tomato Plants

An untargeted metabolomic analysis (UHPLC/QTOF-MS) was performed to understand the molecular mechanisms triggered by PH treatments in tomato and lettuce plants grown under either control or salinity conditions. The untargeted profiling allowed to putatively annotate more than 2,000 compounds; the whole list of metabolites, together with the composite mass spectra and individual abundances are provided as Supplementary Material (Supplementary Table 5A for lettuce, Supplementary Table 5B for tomato). The metabolomics dataset included a broad biochemical diversity, including metabolites from a large range of metabolic processes of primary and secondary metabolism. Multivariate statistics were applied to the metabolomic dataset highlighting the similarities or dissimilarities among the metabolomic profiles across treatments. At first, the unsupervised and supervised statistics were carried out separately, while considering the metabolomics datasets from lettuce and tomato. These statistics served as the first step of interpretation to point out the similarities or dissimilarities among all treatments.

**FIGURE 5:**
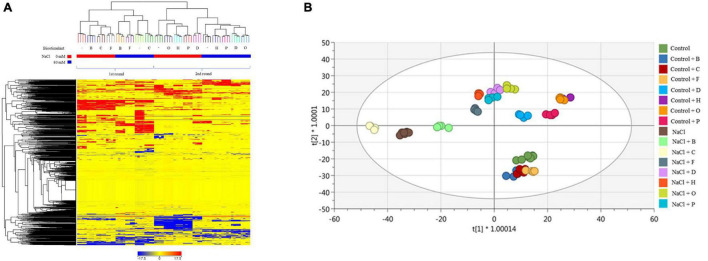
Metabolomic analysis of lettuce plants. Unsupervised hierarchical cluster analysis carried out from ultra-high-pressure liquid chromatographic system (UHPLC-ESI/QTOF-MS) metabolomics analysis of lettuce plants treated with 7 PHs under control and salt stress (NaCl) conditions. The fold-change-based heat map was used to build hierarchical clusters (linkage rule: Ward, distance: Euclidean) **(A)**. Score plot of orthogonal projection to latent structures discriminant analysis (OPLS-DA) supervised modelling carried out on untargeted metabolomics profiles of lettuce plants after 7 PHs application, under control and salt stress (NaCl) conditions (R2Y = 0.98, Q2Y = 0.93) **(B)**.

When the lettuce plants were analysed, the unsupervised fold-change-based hierarchical clustering output (Figure 5A) naively evidenced that within each trial (first for PHs B, C, F, and second for D, H, O, P), the salinity application was the main clustering factor. Nevertheless, the score plot from the supervised OPLS-DA multivariate modelling (Figure 5B) allowed to efficiently discriminate among the different groups of treatments, whereas control samples from the two different trials merged into the score-plot. The model was validated (*P*-value < 0.001), parameters of the OPLS-DA were excellent (R2Y = 0.983, Q2Y = 0.935), and overfitting was avoided through permutation testing. Therefore, discriminant compounds (VIP score > 1.2 −Supplementary Table 6A) were exported and were considered. Overall, primary and secondary metabolites were equally represented among the VIP discriminants. In more detail, the most represented primary metabolites included carbohydrates, membrane lipids, hormones (mainly brassinosteroids, a cytokinin, and two gibberellins), and electron-carriers (quinol and quinones). Among secondary metabolites, the most represented compounds were phenylpropanoids, alkaloids, and isoprenoids. Exploring the OPLS-DA score plot (Figure 5B), the variants showed a clear distribution through all score spaces with a clear separation between stressed and non-stressed plants treated with the same PHs. Under non-saline conditions, the plants treated with B, C, and F presented metabolomic signatures similar to the untreated control, depicting a separated group of responses. However, a second group formed by the plants treated with H, O, and P, corresponding to the best performing biostimulants according to the phenotyping data, formed an independent group under control and salt stress conditions (Figure 5B).

**FIGURE 6:**
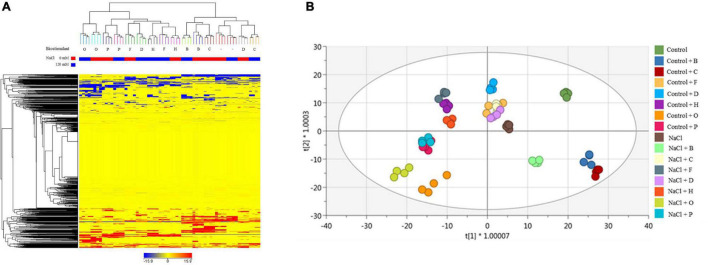
Metabolomic analysis of tomato plants. Unsupervised hierarchical cluster analysis carried out from UHPLC-ESI/QTOF-MS metabolomics analysis of tomatoes treated with 7 PHs under control and salt stress (NaCl) conditions. The fold-change-based heat map was used to build hierarchical clusters (linkage rule: Ward, distance: Euclidean) **(A)**. Score plot of orthogonal projection to latent structures discriminant analysis (OPLS-DA) supervised modelling carried out on untargeted metabolomics profiles of lettuce plants after 7 PHs application, under control and salt stress (NaCl) conditions (R2Y = 0.98, Q2Y = 0.94) **(B)**.

Different results were obtained in tomato plants. As a preliminary approach, unsupervised HCA (Figure 6A) suggests that salinity did not have a primary effect on metabolic signatures. Tomato samples clustered in two main groups, distinguishing PH treatments, with a cluster including O, P, F, and H, and a second group containing those plants treated with the substance B and C, more similar to untreated controls. These results were further confirmed by the OPLS-DA supervised statistics which allowed separating better the samples in the score space according to the combined treatments (Figure 6B). The model parameters of the OPLS-DA were excellent (R2Y = 0.981, Q2Y = 0.941), validation was adequate (*P*-value <0.001), and overfitting could be excluded by permutation testing. Discriminant compounds (VIP score > 1.1 – Supplementary Table 6B) are mainly related to secondary metabolism (phenylpropanoids and to a lesser extent terpenoids and alkaloids), cofactors/electron carriers, and phytohormones (gibberellins, brassinosteroids, jasmonate, and IAA-conjugates). Primary metabolites range from carbohydrates, lipids, organic acids, and nucleic acid components. OPLS-DA evidenced that some PHs (O, P, H, D) were better able to minimize the differences between stressed and non-stressed plants, so the plants were grouped independently of the plant growth conditions. This feature might imply a hierarchically stronger effect of the biostimulant above salt stress, and thus, the ability of these PHs to well-play as stress alleviator on plant metabolism (Figure 6B).

### Biochemical Insights on the Metabolomic Reprogramming Triggered by the Best and the Worst Performers Protein Hydrolysates

**FIGURE 7:**
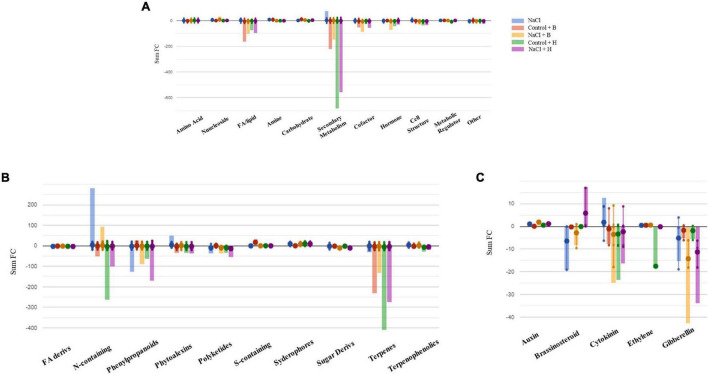
Metabolic changes in lettuce plants. Metabolic processes **(A)**, secondary metabolism **(B)**, and **(C)** hormone biosynthesis were impaired by treatments in lettuce plants compared to control samples. Differential metabolites from the Volcano analysis (*P*-value <0.01, FC ≥ 1.3) were elaborated using the Omics Viewer Dashboard of the Plant Cyc pathway Tool software (www.pmn.plantcyc.com). The large dots represent the average (mean) of all log Fold-change (FC) for metabolites, and the small dots represent the individual log FC for each metabolite. The *x*-axis represents each set of subcategories, while the *y*-axis corresponds to the cumulative log FC. FA/Lipid: fatty acids and lipids, Amine: amines and polyamines, Cofactor: cofactors, prosthetic groups, electron carriers, and vitamins, FA Derives: fatty acid derivatives, N-containing: Nitrogen-containing secondary metabolites, S-containing: Sulfur-containing secondary metabolites, Sugar Derives: sugar derivatives.

**FIGURE 8:**
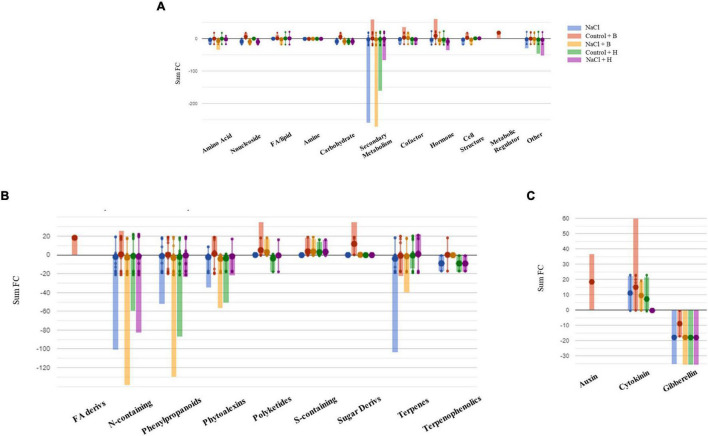
Metabolic changes in tomato plants. Metabolic processes **(A)**, secondary metabolism **(B)**, and **(C)** hormone biosynthesis were impaired by treatments in tomato plants compared to control samples. Differential metabolites from the Volcano analysis (*P*-value <0.01, FC ≥ 1.3) were elaborated using the Omics Viewer Dashboard of the Plant Cyc pathway Tool software (www.pmn.plantcyc.com). The large dots represent the average (mean) of all log Fold-change (FC) for metabolites, and the small dots represent the individual log FC for each metabolite. The *x*-axis represents each set of subcategories, while the *y*-axis corresponds to the cumulative log FC. FA/Lipid: fatty acids and lipids, Amine: amines and polyamines, Cofactor: cofactors, prosthetic groups, electron carriers, and vitamins, FA Derives: fatty acid derivatives, N-containing: Nitrogen-containing secondary metabolites, S-containing: Sulfur-containing secondary metabolites, and Sugar Derives: sugar derivatives.

To further understand the differences between the mode of action of good and bad performing biostimulants, we analysed the plants treated with the best substance, H, as plant growth promotor and stress alleviator and with the worst, B, as growth inhibitor [according to the PBC indexes and the cluster analysis (Figures 3, 4 and Table 1)]. The two corresponding sub-datasets were then considered, including 669 compounds for lettuce and 1,090 for tomato. The HCA confirmed the strongest effect of salinity above the PH-treatments in lettuce, whereas PHs had a hierarchically stronger effect on tomatoes (Supplementary Figures 9A,B). Indeed, for lettuce, the two main clusters divided stressed from non-stressed plants, even though the metabolic profiles of control plants were more similar to B-treated plants. On the other hand, the metabolic signatures of tomato samples merged in two main clusters, one including H-treated plants and a second cluster grouped controls and B-treated. Consistent results were obtained by OPLS-DA where the separation of treatments could be observed in the score plot hyperspace (Supplementary Figures 9A,B). The OPLS-DA model was robust, being R2Y = 0.996 and Q2Y = 0.987 in lettuce (*P*-value < 0.001) and R2Y = 0.996 and Q2Y = 0.977 in tomato (*P*-value < 0.001). Thereafter, the Volcano plot analysis (*P*-value < 0.01, FC ≥ 1.3) was applied to identify differential compounds. Overall, we evidenced that 414 (in lettuce, Supplementary Table 7A) and 261 compounds (in tomato, Supplementary Table 7B) were significantly modulated by treatments compared to control. The Pathway Tool analysis from PlantCyc was applied to simplify the interpretation in terms of plant metabolism. Figures 7A, 8A show the biosynthetic processes modulated by treatments, along with cumulate FC values. Overall, biosynthesis processes related to secondary metabolism were generally decreased in both crops (Figures 7B, 8B), except for tomato plants treated with PH B under non-stress conditions. In both species, N-containing compounds (mostly alkaloids), phenylpropanoids, and terpenes underwent the most evident modulation. In lettuce, several membrane lipids were impaired, such as long-chain fatty acid (also in the epoxy form) and sterols. Phytohormones were broadly affected by the treatments in lettuce, whereas in tomato, we evidenced a weaker impact (Figures 7C, 8C). The main modulations concerned gibberellins, which decreased in both crops. In treated lettuce, a reduction of brassinosteroids, auxin-conjugates (IAA-Ile, IAA-Leu, IAA-Asp), and N-glycosylated cytokinins were observed. The ethylene precursor (1-aminocyclopropane-1-carboxylate, ACC) down-accumulated only in control plants of lettuce treated with H-substance. In tomatoes, changes in cytokinin content with the main accumulation of trans-zeatin-O-glucoside-7-*N*-glucoside in response to PH B (either under control conditions or salt stress and to H in control conditions).

Similar results were obtained when the effect of the two PHs (B and H) was investigated with respect of their ability as growth improvers and stress alleviators by independently exploring non-stressed and salt-stressed plants. Two different OPLS-DA models were validated regardless of the plant species, one considering metabolomics data from salt-stressed plants and the other including non-stressed plants (Supplementary Figures 10A,B). Validation parameters were excellent in both models, showing a R2Y = 0.992 and Q2Y = 0.964 (*P*-value = 1.57 e-17) for non-stressed samples and R2Y = 0.996 and Q2Y = 0.949 (*P*-value = 6.41 e-15) for samples grown under salt stress. The strongest discriminant compounds were selected from each model through the VIP method (VIP score > 1.20). A total of 310 (salinity, Supplementary Table 8A) and 333 (control, Supplementary Table 8B) metabolites were considered and exported along with their FC values into the Omic Viewer Pathway Tool of PlantCyc for interpretations (Supplementary Figures 11A–C, 12A–C). Half of the total discriminant compounds were classified as secondary metabolites. However, whereas B substance downregulated the accumulation of secondary metabolites (phenylpropanoids, terpenes, and N-containing compounds), H increased them along with the levels of others such as fatty acid/lipids, cofactors, and electron carriers.

Regarding phytohormones, several discriminant compounds were differentially modulated by the two PHs. Under non-stress conditions, brassinosteroids [3-dehydroteasterone and (22S,24R)-22-hydroxy-5α-ergostan-3-one] strongly down-accumulated in response to PH B but not to PH H, which, on the other hand, remarkably induced a strong accumulation of methyl (indol-3-yl) acetate (MeIAA), a storage form of IAA. Among cytokinins, two glycosylated forms of trans-zeatin accumulated by H applications, whereas only one (*trans*-zeatin-7-*N*-glucoside) in response to B. The PH-treated plants caused a depletion in the ethylene precursor [1-aminocyclipropane-1-carboxylate (ACC)], but PH H had the strongest effect. Under salinity conditions, MeIAA showed the same modulations recorded in control plants. The only cytokinin found as discriminant (cis-zeatin) accumulated in response to the substance H.

### Integrative Analysis of Phenomic and Metabolomics Datasets

The multivariate generalisation of the squared Pearson correlation coefficient was investigated through co-inertia (CIA) in terms of global similarity between the integrated phenotyping and metabolomic datasets (Supplementary Figure 13). The overall correlation between the two datasets was expressed as the RV coefficient. This is a measure of global similarity between the datasets and assumes values between 0 and 1. The closer to 1, the higher the similarity between the datasets (Robert and Escoufier, 1976). The overall similarity in structure between phenotyping and metabolomics data was higher in lettuce than in tomato with an RV coefficient equal to 0.37 and 0.29, respectively. However, the obtained RV for both crops reflected the lack of joint structure in these two datasets (phenomics and metabolomics). Altogether, we could say that according to the low synchrony obtained between the phenotypical and metabolomics data after CIA analysis, the changes in the metabolic content do not define the phenotype of the plants.

To deal with this low concordance between the two datasets, we decided to work with the phenotypical and metabolomic data obtained from the plants treated with the substance H as plant growth promotor and stress alleviator, and with the substance B that worked as a growth inhibitor in both lettuce and tomato plants. As the first step, we used the random forest classification method to identify the most important phenotyping traits for each species. As a result, in lettuce, the importance was mainly focused on morphological traits, whereas in tomato the physiology was most relevant (Supplementary Tables 4A,B). Concretely, the volume represented as DB was the parameter with the highest discriminative power between treatments, followed by the physiological parameter, water use efficiency (WUE), related to water balance. However, in tomatoes, the most important parameters were related to the photosynthetic performance of the plant, with QY_max and QY_Lss4 as the main ones. The two crops respond in a different way to the changes in the growth conditions, where not only the growth conditions but also whatever treatment applied is included. Once defined, the main phenotypical traits, the correlated metabolites (*p* < 0.05) were identified performing a correlation matrix (Supplementary Tables 9A,B). For lettuce, many secondary metabolites, including alkaloids, terpenoids, and phenols or certain metabolites involved in amino acid metabolism (mainly degradation compounds) were negatively correlated with the volume of lettuce plants. However, this phenotyping trait was positively correlated with IAA, IAA-Asp, and L-arginine-succinate, among others. In tomato, however, QY_max was positively correlated to certain secondary metabolites, such as the phenol 4-hydroxycoumarin, and the vitamin K1 (phylloquinone), among others, and the carbohydrate D-erythrose 4-phosphate.

## Discussion

In the last years, the use of plant phenotyping approaches is becoming an efficient tool for characterizing the mode of action of biostimulants obtained from many different sources and in many plant species (Briglia et al., 2019; Danzi et al., 2019; Akhtar et al., 2020; Mutale-joan et al., 2020). Non-invasive approaches allow the simultaneous study of the crops grown under different growth conditions treated with biostimulant substances for a better understanding of their mode of action. To this end, our study could be another example of this type of study. We show that two distance crops, such as lettuce and tomato, differ in the response to salt stress alone or the interaction between the stress and the application of PHs based biostimulants. The PH application modified the kinetics of the curves for the different phenotyping traits, including plant growth, fluorescence-related parameters, and thermal imaging. This separated the plant response in the early and late phases, with it being more evident for lettuce than for tomato plants (Supplementary Figures 1–8). However, the effect was different for both crops and from the one obtained in previous studies performed in Arabidopsis (Sorrentino et al., 2021). Whereas in lettuce, the biostimulant application induced changes during the early phase and after a low number of applications, in the case of tomato the changes were mainly visible at the late phase.

To go further in the understanding of the biostimulant mode of action, we probed the combination of phenotyping experiments with other omics, especially metabolomics, which can give additional information. In this context, the most difficult part is the data management, as both –omics approaches are ending with a huge amount of data to process and interpret. Thanks to the fast evolution of the data analysis based on multivariate statistical analysis, this is possible, and this aims to be a good example of such approaches. For that, the first step done was the clustering of the variants analysed independently for both lettuce and tomato using phenomic data (Figure 4). The high dimensionality of the data is a characteristic that creates many challenges in clustering and data analysis in general. The clustering tree is defined by the analysis of the L_K_ norm distances that depend on the value of K (Euclidean, Manhattan, Minkowski, etc.). The most often L_k_ norm used is Euclidean distance. In this regard, Aggarwal et al. (2001) showed some interesting results comparing different L_K_ norm distances. More specifically, they stress that the meaningfulness of L_K_ norm (K = 1 for Manhattan, K = 2 for Euclidean, etc.) is worse on high dimensions. This means that the Manhattan distance is preferred in situations where the number of traits (metabolomics or phenomics) is considerably large. That is the reason why Euclidean and Manhattan distances were both examined in this study. However, in this case, the results were not significantly different for both lettuce and tomato. One of the reasons for this result could be that there was a clear different response of the plant when the H or B substance was applied.

As the second step, after the performance of the metabolic analysis and data processing, the concordance between both datasets (phenomics + metabolomics) was performed using CIA analysis. This tool is becoming a particularly attractive method for the identification of relationships between large datasets, but it is mainly used in ecology or genetics (Bady et al., 2004; Genitsaris et al., 2016; Devarajan et al., 2021). However, there are not any case studies using this tool for integrating phenotyping data with other omics. In our study, we observed low values of the RV coefficient between both datasets (phenomics + metabolomics) (Supplementary Figure 13). This would mean that the metabolic profiling cannot explain the phenotypes of the plants, making the integration of both data more difficult. One of the reasons for this could be that the most of modulated metabolites were secondary metabolites, including alkaloids, phenylpropanoids, and terpenes (Figures 7, 8 and Supplementary Table 7). On the contrary, relevant molecules, such as plant growth regulators, are not so abundant and mainly appeared in lettuce. For example, in lettuce plants, there was a clear reduction of the conjugated forms of IAA, most probably to maintain the pool of IAA and thus allow the plant growth (reviewed by Ludwig-Müller, 2011). Besides, the precursor of ethylene, ACC, was also reduced in lettuce plants treated with the H substance when plants were grown under control conditions. It could mean that the H application can reduce the ethylene synthesis and with that, its negative effects (i.e., growth inhibition). However, recent studies also showed that ACC itself is enough to reduce the plant growth (Vanderstraeten et al., 2019).

To solve the low concordance between the phenotyping and metabolomic data, we decided to identify the most significant traits for each treatment (or treatment + biostimulant) among the phenotyping traits identified. For that, we used a random forest classifier. Such a tool is mainly used in plant science for machine learning approaches applied in image analysis (Barradas et al., 2021; Singh et al., 2016), but it has never been used for characterizing the biostimulant mode of action. Apart from a powerful classification method, the random forest has the advantage of revealing the significance of the traits used for identifying (classifying) treatments. This is done using the decrease in classification accuracy if a specific variable – trait is removed. The random forest classifiers applied for lettuce and tomato have high accuracy percentages (>95%), which makes them valid for the interpretation of the significant traits. The significant traits found for lettuce were the volume (based on the DB) and WUE. The most relevant result was the volume that positively correlated with the IAA levels (Supplementary Table 9). Higher IAA levels in leaves can improve cell extensibility and consequently induce leaf growth (Veselov et al., 2002). Additionally, under stress conditions, the IAA accumulation can be a stress tolerance mechanism that permits the plant to keep growing (De Diego et al., 2012). Besides, this result could also explain the aforementioned reduction of the conjugation of IAA with certain amino acids observed in the plants treated with the H substance, and hence, their better growth under both control and stress conditions. The amide-linked IAA-amino acid conjugates are considered reversible storage forms with no or low biological activity (Mellor et al., 2016), with the Gretchen Hagen3 (GH3) family of auxin-inducible acyl amido synthetases as the enzymes converting IAA to IAA-amino acids. Thus, we could think that the application of the substance H in lettuce has downregulated the activity of GH3 to reduce auxin-conjugates and maintain the IAA levels as a stress response strategy.

In tomato, the most important trait was the QY_max, which was positively correlated to D-erythrose 4-phosphate (Supplementary Table 9), an intermediate in the pentose phosphate pathway, and the Calvin cycle that serves as a precursor in the shikimate pathway (Billakurthi and Schreier, 2020). This result could also explain the positive correlation with other metabolites product of this pathway, such as 4-hydroxycoumarin and vitamin K1 (phylloquinone). The hydroxycoumarins have been described as efficient antibacterial compounds that can improve plant stress resistance (Yang et al., 2018). Vitamin K1 has been detected inside thylakoid membranes as an electron carrier and is a key element within the photosystem I redox chain (reviewed by Lüthje et al., 2013). Thus, it serves as a mobile carrier transferring the electrons across the plasma membrane and contributes to the maintenance of a suitable redox state of some important proteins embedded in the plasma membrane with protective functions against stress. The better performance in tomato plants can, thus, be related to the use of D-erythrose 4-phosphate as a precursor for the synthesis of antistress compounds from the shikimate pathway.

## Conclusion

We assume that PH-based biostimulants improve the plant growth and salt stress response in crops, such as lettuce and tomato, through different mechanisms. For a better understanding of the mechanism of action, it was necessary to use powerful statistical tools which helped to simplify the results and, hence, their interpretation. Thus, we observed that for lettuce, the most interesting traits to study the PHs based biostimulants are those representing the aerial biomass (i.e., volume). These were correlated with altered levels of certain phytohormones, such as auxin and ethylene, and consequently with plant growth. However, in tomatoes, the chlorophyll fluorescence-related parameters were the most relevant defining the plant growth capacity and salt stress tolerance affecting also the stress-related metabolites from the shikimate pathway. We believe that these results corroborated the relevant role of the multivariate statistical analysis as a further step to uncover key traits and metabolites for a deeper understanding of the biostimulant mode of action.

## Data Availability Statement

The original contributions presented in the study are included in the article/Supplementary Material, further inquiries can be directed to the corresponding authors.

## Author Contributions

YR, LL, and GC prepared and selected the protein hydrolysates. MS and KP designed the phenotyping experiments. MS performed the experiments, the image processing, and image-based data analysis. LL, VB, and PG carried out the untargeted metabolomics and performed the analysis of the metabolomic data. IS and ND performed the multivariate statistical analysis. All authors discussed the results and contributed to writing the manuscript.

## Conflict of Interest

MS and KP were employed by company Photon Systems Instruments (PSI). The remaining authors declare that the research was conducted in the absence of any commercial or financial relationships that could be construed as a potential conflict of interest.

## Publisher’s Note

All claims expressed in this article are solely those of the authors and do not necessarily represent those of their affiliated organizations, or those of the publisher, the editors and the reviewers. Any product that may be evaluated in this article, or claim that may be made by its manufacturer, is not guaranteed or endorsed by the publisher.

## Footnotes

1
http://www.plantcyc.org

## Supplementary Material

Supplementary Figure 1 Morphological and physiological parameters of lettuce plants under control and stress conditions. Digital biomass [DM, **(A1–B1)**], roundness **(A2–B2)**, compactness **(A3–B3)**, and slenderness of leaves [SOL, **(A4–B4)**] in lettuce plants grown under control or salt stress conditions for 21 days of phenotyping (DoP). Variations of maximum quantum yield of PSII photochemistry for the light-adapted state [Fv′/Fm′, **(C1–D1)**], PSII operating efficiency [ΦPSII, **(C2–C3)**] and non-photochemical quenching [NPQ, **(C3–D3)**]. The values were obtained after the exposure of the plants to a light intensity of 480 μmol m^–2^ s^–1^ (Lss2). Morphological and physiological values shown represent the average of 8 biological replicates per variant. Error bars represent standard deviation. The significant differences between control and salt treatment are indicated with *, ^**^, and ^***^ for *p*-values below 0.05, 0.01, and 0.001, respectively.

Supplementary Figure 2 Morphological and physiological parameters of tomato plants under control and stress conditions. Digital biomass [DB, **(A1)**], roundness **(A2)**, compactness **(A3)**, and slenderness of leaves [SOL, **(A4)**] in tomato plants grown under control or salt stress conditions for 24 days of phenotyping (DoP). The maximum quantum yield of PSII photochemistry for the light-adapted state [Fv′/Fm′, **(C1)**], PSII operating efficiency [ΦPSII, **(C2)**], and non-photochemical quenching [NPQ, **(C3)**]. The values were obtained after the exposure of the plants to a light intensity of 480 μmol m^–2^ s^–1^ (Lss2). Morphological and physiological values shown represent the average of 6 biological replicates per variant. Error bars represent standard deviation. The significant differences between control and salt treatment are indicated with *, ^**^, and ^***^ for *p*-values below 0.05, 0.01, and 0.001, respectively.

Supplementary Figure 3 Morphological and physiological parameters of lettuce plants treated with 7 different under control conditions. Digital biomass [DB, **(A1–B1)**], roundness **(A2–B2)**, compactness **(A3–B3)**, and slenderness of leaves [SOL, **(A4–B4)**] of lettuce plants treated with 7 PHs and grown under control conditions for 21 days of phenotyping (DoP). The maximum quantum yield of PSII photochemistry for the light-adapted state [Fv′/Fm′, **(C1–D1)**], PSII operating efficiency [ΦPSII, **(C2–C3)**] and non-photochemical quenching [NPQ, **(C3–D3)**]. The values were obtained after the exposure of the plants to a light intensity of 480 μmol m^–2^ s^–1^ (Lss2). Morphological and physiological values shown represent the average of 8 biological replicates per variant. Error bars represent standard deviation.

Supplementary Figure 4 Morphological and physiological parameters of lettuce plants treated with 7 different under salt stress conditions. Digital biomass [DM, **(A1–B1)**], roundness **(A2–B2)**, compactness **(A3–B3)**, and slenderness of leaves [SOL, **(A4–B4)**] of lettuce plants treated with 7 PHs and grown under salt stress conditions for 21 days of phenotyping (DoP). The maximum quantum yield of PSII photochemistry for the light-adapted state [Fv′/Fm′, **(C1–D1)**], PSII operating efficiency [ΦPSII, **(C2–C3)**] and non-photochemical quenching [NPQ, **(C3–D3)**]. The values were obtained after the exposure of the plants to a light intensity of 480 μmol m^–2^ s^–1^ (Lss2). Morphological and physiological values shown represent the average of 8 biological replicates per variant. Error bars represent standard deviation.

Supplementary Figure 5 Morphological and physiological parameters of tomato plants grown in control and salt stress conditions: PHs treated and untreated lettuce plants. Digital biomass [DM, **(A1–B1)**], roundness **(A2–B2)**, compactness **(A3–B3)**, and slenderness of leaves [SOL, **(A4–B4)**] of tomato plants treated with 7 PHs and grown under control and salt stress conditions for 24 days of phenotyping (DoP). The maximum quantum yield of PSII photochemistry for the light-adapted state [Fv′/Fm′, **(C1–D1)**], PSII operating efficiency [ΦPSII, **(C2–C3)**] and non-photochemical quenching [NPQ, **(C3–D3)**]. Values were obtained after the exposure of the plants to the light of intensity 480 μmol m^–2^ s^–1^ (Lss2). Morphological and physiological values shown represent the average of 6 biological replicates per variant. Error bars represent standard deviation. The significant differences between control and salt treatment are indicated with *, ^**^, and ^***^ for *p*-values below 0.05, 0.01, and 0.001, respectively.

Supplementary Figure 6 Relative Growth Rate and final biomass of lettuce plants treated with PHs. Relative Growth Rate [RGR, **(A1–A2)**] of the different treatments over time, calculated for the early phase (from DoP 0 to DoP 12) and for the late Phase (from DoP 12 to DoP 21) in lettuce plants treated with 7 PHs grown under control or salt stress conditions. Total fresh **(B1–B2)** and dry **(C1–C2)** weight of the final aboveground biomass per variant. Values represent the average of the 8 biological replicates per variant. Error bars represent standard deviation. Different letters indicate significant differences according to the one-way ANOVA post hoc Tukey’s test (*p* < 0.05).

Supplementary Figure 7 Relative Growth Rate and final biomass of tomato plants treated with PHs. Relative Growth Rate [RGR, **(A1–A2)**] of the different treatments over time, calculated for the early phase (from DoP 0 to DoP 12) and for the late Phase (from DoP 12 to DoP 24) in tomato plants sprayed with 7 PHs, grown under control conditions or salt stress. Total fresh **(B1–B2)** and dry **(C1–C2)** weight of the final aboveground biomass per variant. Values represent the average of the 6 biological replicates per variant. Error bars represent standard deviation. Different letters indicate significant differences according to the one-way ANOVA post hoc Tukey’s test (*p* < 0.05).

Supplementary Figure 8 Temperature of the leaves for lettuce and tomato plants under control and salt stress conditions. Canopy temperature depression measured on lettuce [**(A1–A3, B1–B3)**, full and dotted lines] and tomato [**(C1–C3)**, dashed and dotted + dashed lines] plants, untreated or treated with the biostimulant substances, grown under control or salt stress conditions. Values represent the average of the 8 biological replicates per treatment in lettuce and 6 biological replicates in tomato, error bars represent standard deviation. The significant differences between control and salt treatment are indicated with *, ^**^, and ^***^ for *p*-values below 0.05, 0.01, and 0.001, respectively.

Supplementary Figure 9 Metabolomic analysis for the best (H) and worst (B) performing biostimulants. Unsupervised hierarchical cluster analysis carried out from UHPLC-ESI/QTOF-MS metabolomics analysis of lettuce **(A)** and tomato **(B)** plants treated with PH B or H, grown under control or salt stress conditions. The fold-change-based heat map was used to build hierarchical clusters (linkage rule: Ward, distance: Euclidean). Score plot of orthogonal projection to latent structures discriminant analysis (OPLS-DA) supervised modelling carried out on untargeted metabolomics profiles of lettuce **(C)** and tomato **(D)** plants after B and H application grown under control and salt stress (NaCl) condition.

Supplementary Figure 10 Score plot of metabolomics profiles. Score plot orthogonal projection to latent structures discriminant analysis (OPLS-DA) supervised modelling carried out on untargeted metabolomics profiles of tomato and lettuce plants after B and H application grown under control **(A)** or salt stress **(B)** conditions.

Supplementary Figure 11 Identified metabolites in control conditions. Metabolic processes **(A)**, secondary metabolism **(B)**, and hormone biosynthesis **(C)** were impaired by treatments in lettuce and tomato plants grown under control conditions. Differential metabolites (VIP score > 1.20) along with their fold-change (FC) values were elaborated using the Omic Viewer Dashboard of the PlantCyc pathway Tool software (www.pmn.plantcyc.com). The large dots represent the average (mean) of all log FC for metabolites, and the small dots represent the individual log FC for each metabolite. The *x*-axis represents each set of subcategories, while the *y*-axis corresponds to the cumulative log FC. FA/Lipid: fatty acids and lipids, Amine: amines and polyamines, Cofactor: cofactors, prosthetic groups, electron carriers, and vitamins, N-containing: Nitrogen-containing secondary metabolites, S-containing: Sulphur-containing secondary metabolites, Sugar Derives: sugar derivatives.

Supplementary Figure 12 Identified metabolites in plant under salt stress conditions. Metabolic processes **(A)**, secondary metabolism **(B)**, and hormone biosynthesis **(C)** were impaired by treatments in lettuce and tomato plants grown under salt stress conditions. Differential metabolites (VIP score > 1.20) along with their fold-change (FC) values were elaborated using the Omic Viewer Dashboard of the PlantCyc pathway Tool software (https://plantcyc.org/). The large dots represent the average (mean) of all log FC for metabolites, and the small dots represent the individual log FC for each metabolite. The *x*-axis represents each set of subcategories, while the *y*-axis corresponds to the cumulative log FC. FA/Lipid: fatty acids and lipids, Amine: amines and polyamines, Cofactor: cofactors, prosthetic groups, electron carriers, and vitamins, N-containing: Nitrogen-containing secondary metabolites, S-containing: Sulphur-containing secondary metabolites, and Sugar Derivs: sugar derivatives.

Supplementary Figure 13 Graphical output of co-Inertia analysis (CIA). Scatter plot of tomato **(A)** and lettuce **(B)** samples. Each sample is represented by an arrow whose length is proportional to the divergence between the phenomic and the metabolomic datasets. Eigenvalues of the co-inertia analysis for tomato **(C)** and lettuce **(D)**. Correlation circles **(E,F)** showing the projections of the PCA axes (from the phenomic datasets) onto the axes of the co-inertia analysis (*x* axes) and projections of the PCA axes (from the metabolomic datasets) onto the axes of the co-inertia analysis (*y* axes). These four circles represent a view of the rotations needed to associate the two datasets for tomato **(E)** and the two datasets for lettuce **(F)**.

Supplementary Table 1 Pairwise comparisons test using mixed models for determining the significant differences between the means of the morphological parameters in lettuce (1st round, A, 2nd round, B) and tomato (C) plants treated with 7 PHs grown under control or salt stress conditions at different time points. The *p*-values below 0.05 are highlighted in green.

Supplementary Table 2 Pairwise comparisons test using mixed models for determining the significant differences between the means of the photosynthetic parameters in lettuce (1st round, A, 2nd round, B) and tomato (C) plants treated with 7 PHs grown under control or salt stress conditions at different time points. The *p*-values below 0.05 are highlighted in green.

Supplementary Table 3 Pairwise comparisons test using mixed models for determining the significant differences between the means of the difference between the canopy and air temperature in lettuce (1st round, A, 2nd round, B) and tomato (C) plants treated with 7 PHs grown under control or salt stress conditions at different time points. The *p*-values below 0.05 are highlighted in green.

Supplementary Table 4 Importance of variables for lettuce (A) and tomato (B) plants based on the random forest classifier used for treatment classification based on a mean decrease in classification accuracy measure.

Supplementary Table 5 Whole dataset produced from untargeted metabolomics carried out in lettuce (A) and tomato (B) plants treated with 7 PHs grown under control or salt stress conditions. Compounds are presented with individual intensities and composite mass spectra.

Supplementary Table 6 Discriminant metabolites identified by the variable importance in projection (VIP) analysis following OPLS-DA modelling of the metabolome in lettuce (A) and tomato (B) plants treated with 7 PHs grown under control or salt stress condition. Compounds were selected as discriminant by possessing a VIP score > 1.20.

Supplementary Table 7 Differential metabolites derived from Volcano analysis (*p* < 0.01, FC ≥ 1.3) in lettuce (A) and tomato (B) plants treated with PH B, and PH H grown under control or salt stress conditions.

Supplementary Table 8 Discriminant metabolites identified by the variable importance in projection (VIP) analysis following OPLS-DA modelling of the metabolome of lettuce and tomato plants treated with PH H and PH B grown under control (A) or salt stress (B) conditions along with their LogFC values in comparison to control samples. Compounds were selected as discriminant by possessing a VIP score > 1.20.

Supplementary Table 9 Correlation matrix between the most important phenotyping traits (according to the random forest analysis) and the metabolites for lettuce (A) and tomato plants (B).
