# Supplementary figures and images for: Integration of Phenomics and Metabolomics Datasets Reveals Different Mode of Action of Biostimulants Based on Protein Hydrolysates in Lactuca sativa L. and Solanum lycopersicum L. Under Salinity
Source: Front Plant Sci. 2022 Feb 3;12:808711. doi: 10.3389/fpls.2021.808711 (PMC8851396; doi:10.3389/fpls.2021.808711)

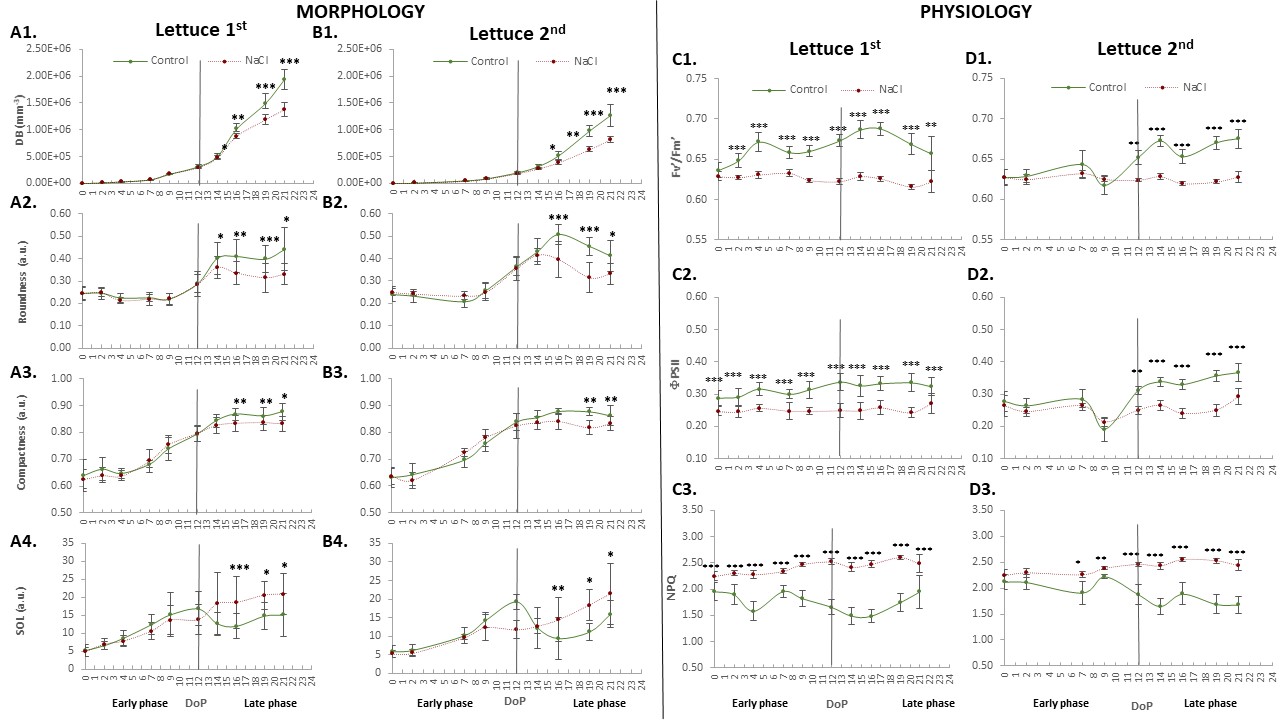

Supplement: Supplementary Figure 1 — Morphological and physiological parameters of lettuce plants under control and stress conditions. Digital biomass [DM, (A1–B1)], roundness (A2–B2), compactness (A3–B3), and slenderness of leaves [SOL, (A4–B4)] in lettuce plants grown under control or salt stress conditions for 21 days of phenotyping (DoP). Variations of maximum quantum yield of PSII photochemistry for the light-adapted state [Fv′/Fm′, (C1–D1)], PSII operating efficiency [ΦPSII, (C2–C3)] and non-photochemical quenching [NPQ, (C3–D3)]. The values were obtained after the exposure of the plants to a light intensity of 480 μmol m–2 s–1 (Lss2). Morphological and physiological values shown represent the average of 8 biological replicates per variant. Error bars represent standard deviation. The significant differences between control and salt treatment are indicated with *, **, and *** for p-values below 0.05, 0.01, and 0.001, respectively. [file Image_1.JPEG]

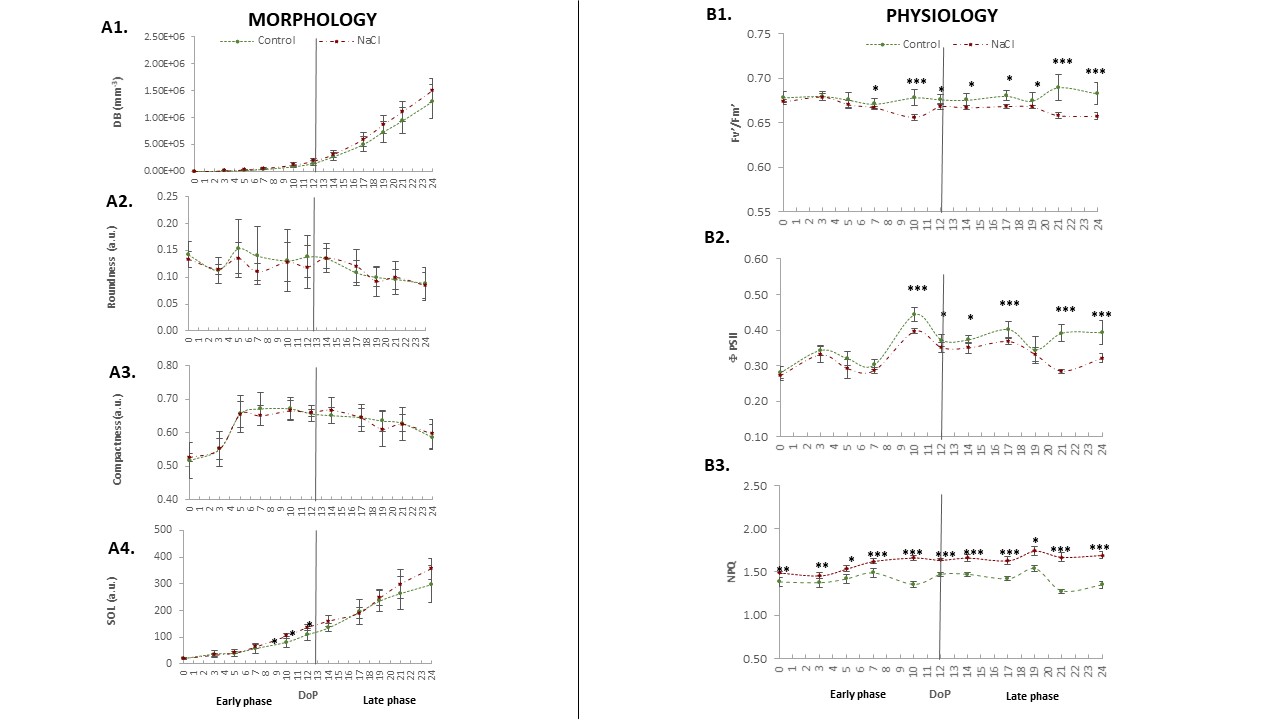

Supplement: Supplementary Figure 2 — Morphological and physiological parameters of tomato plants under control and stress conditions. Digital biomass [DB, (A1)], roundness (A2), compactness (A3), and slenderness of leaves [SOL, (A4)] in tomato plants grown under control or salt stress conditions for 24 days of phenotyping (DoP). The maximum quantum yield of PSII photochemistry for the light-adapted state [Fv′/Fm′, (C1)], PSII operating efficiency [ΦPSII, (C2)], and non-photochemical quenching [NPQ, (C3)]. The values were obtained after the exposure of the plants to a light intensity of 480 μmol m–2 s–1 (Lss2). Morphological and physiological values shown represent the average of 6 biological replicates per variant. Error bars represent standard deviation. The significant differences between control and salt treatment are indicated with *, **, and *** for p-values below 0.05, 0.01, and 0.001, respectively. [file Image_2.JPEG]

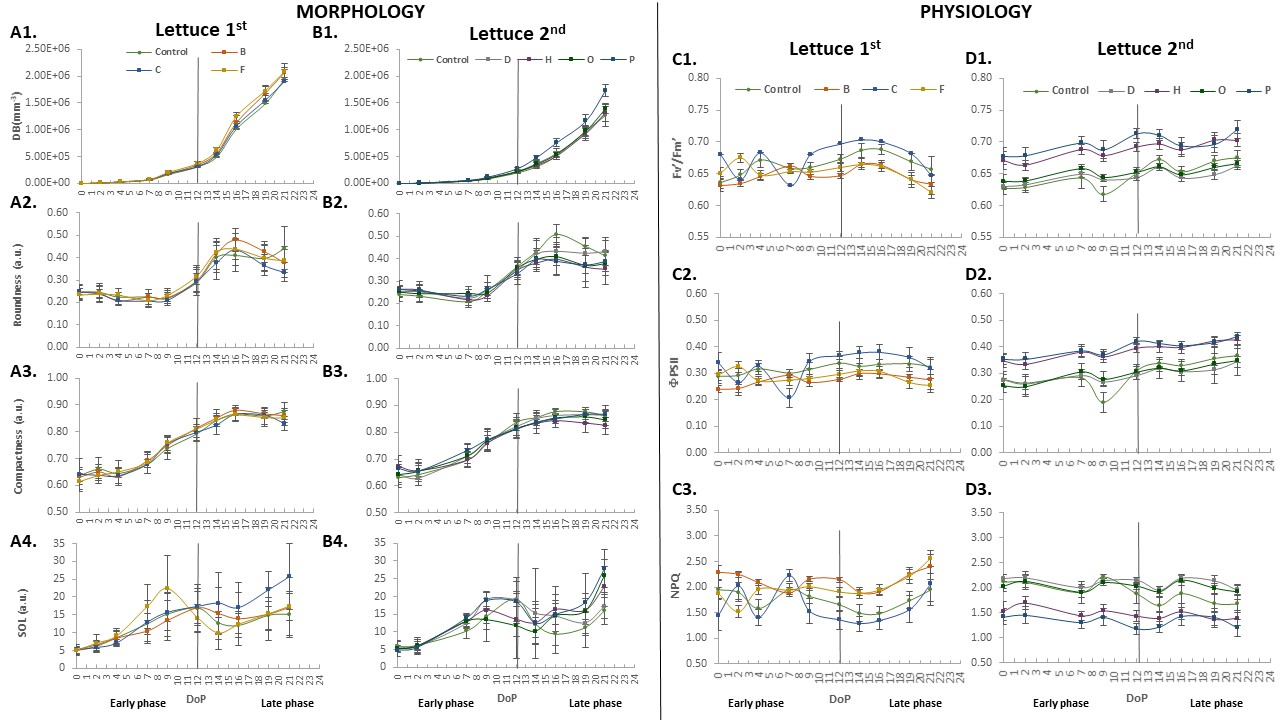

Supplement: Supplementary Figure 3 — Morphological and physiological parameters of lettuce plants treated with 7 different under control conditions. Digital biomass [DB, (A1–B1)], roundness (A2–B2), compactness (A3–B3), and slenderness of leaves [SOL, (A4–B4)] of lettuce plants treated with 7 PHs and grown under control conditions for 21 days of phenotyping (DoP). The maximum quantum yield of PSII photochemistry for the light-adapted state [Fv′/Fm′, (C1–D1)], PSII operating efficiency [ΦPSII, (C2–C3)] and non-photochemical quenching [NPQ, (C3–D3)]. The values were obtained after the exposure of the plants to a light intensity of 480 μmol m–2 s–1 (Lss2). Morphological and physiological values shown represent the average of 8 biological replicates per variant. Error bars represent standard deviation. [file Image_3.JPEG]

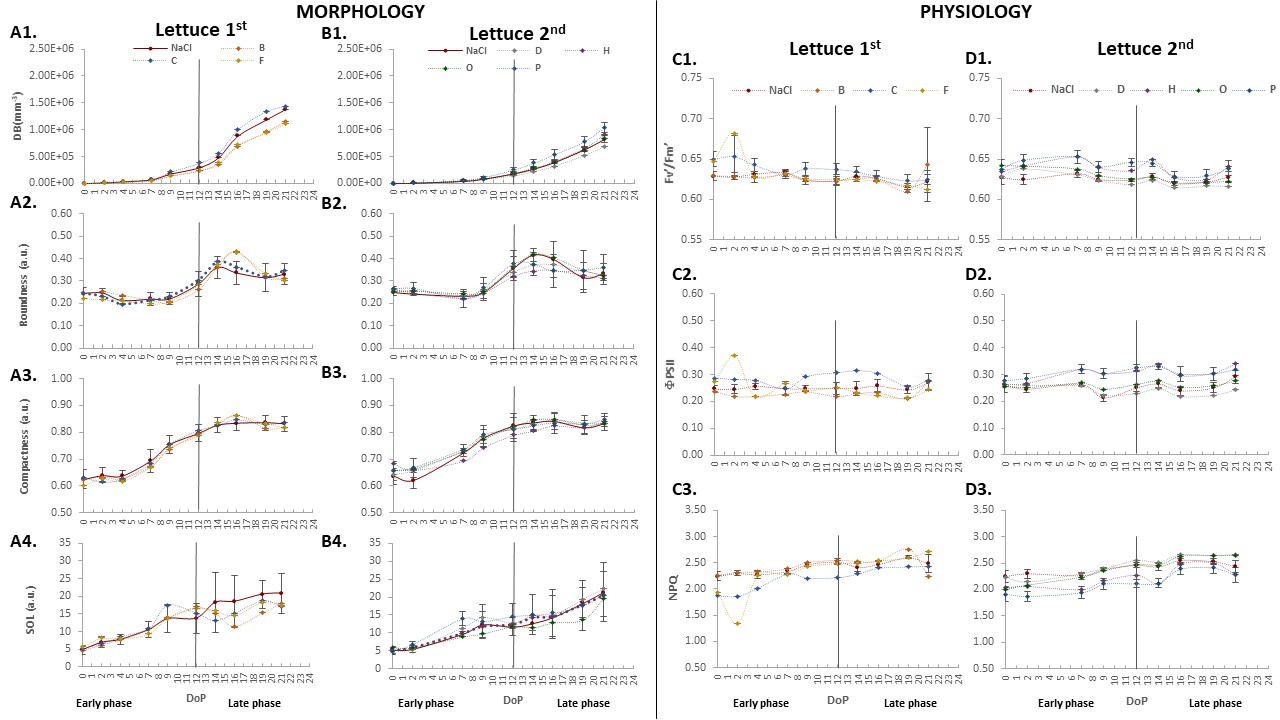

Supplement: Supplementary Figure 4 — Morphological and physiological parameters of lettuce plants treated with 7 different under salt stress conditions. Digital biomass [DM, (A1–B1)], roundness (A2–B2), compactness (A3–B3), and slenderness of leaves [SOL, (A4–B4)] of lettuce plants treated with 7 PHs and grown under salt stress conditions for 21 days of phenotyping (DoP). The maximum quantum yield of PSII photochemistry for the light-adapted state [Fv′/Fm′, (C1–D1)], PSII operating efficiency [ΦPSII, (C2–C3)] and non-photochemical quenching [NPQ, (C3–D3)]. The values were obtained after the exposure of the plants to a light intensity of 480 μmol m–2 s–1 (Lss2). Morphological and physiological values shown represent the average of 8 biological replicates per variant. Error bars represent standard deviation. [file Image_4.JPEG]

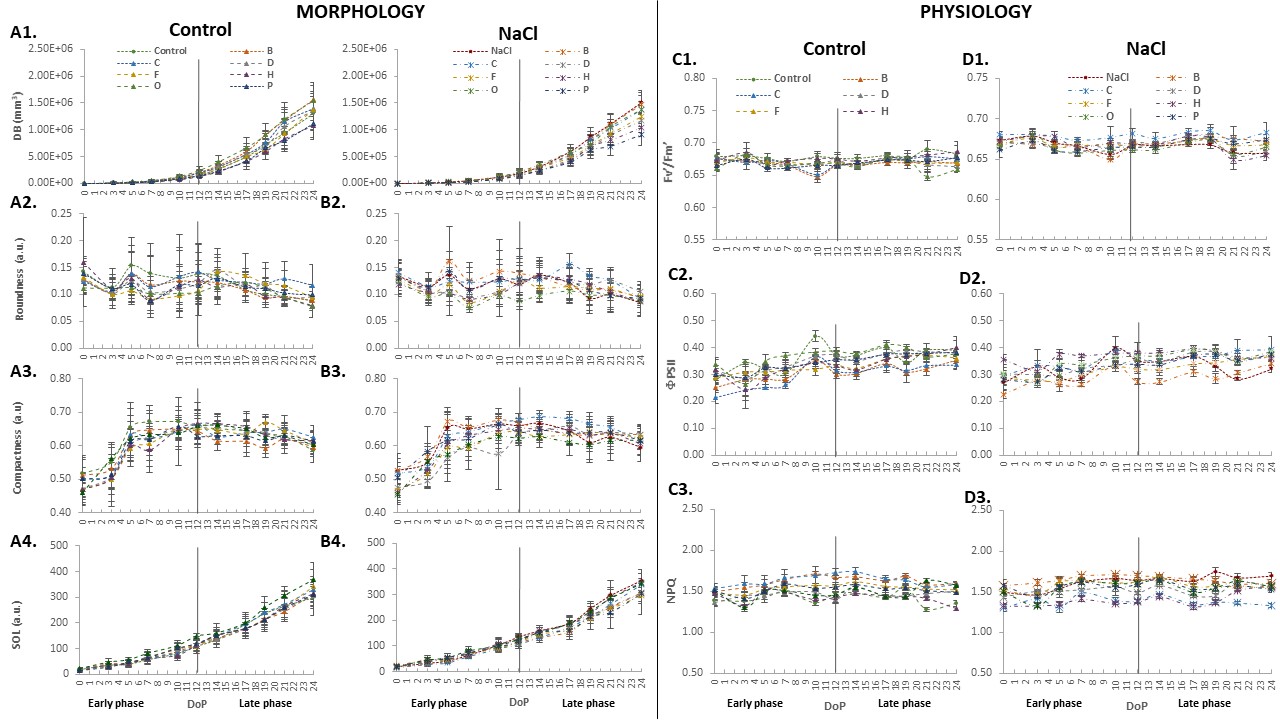

Supplement: Supplementary Figure 5 — Morphological and physiological parameters of tomato plants grown in control and salt stress conditions: PHs treated and untreated lettuce plants. Digital biomass [DM, (A1–B1)], roundness (A2–B2), compactness (A3–B3), and slenderness of leaves [SOL, (A4–B4)] of tomato plants treated with 7 PHs and grown under control and salt stress conditions for 24 days of phenotyping (DoP). The maximum quantum yield of PSII photochemistry for the light-adapted state [Fv′/Fm′, (C1–D1)], PSII operating efficiency [ΦPSII, (C2–C3)] and non-photochemical quenching [NPQ, (C3–D3)]. Values were obtained after the exposure of the plants to the light of intensity 480 μmol m–2 s–1 (Lss2). Morphological and physiological values shown represent the average of 6 biological replicates per variant. Error bars represent standard deviation. The significant differences between control and salt treatment are indicated with *, **, and *** for p-values below 0.05, 0.01, and 0.001, respectively. [file Image_5.JPEG]

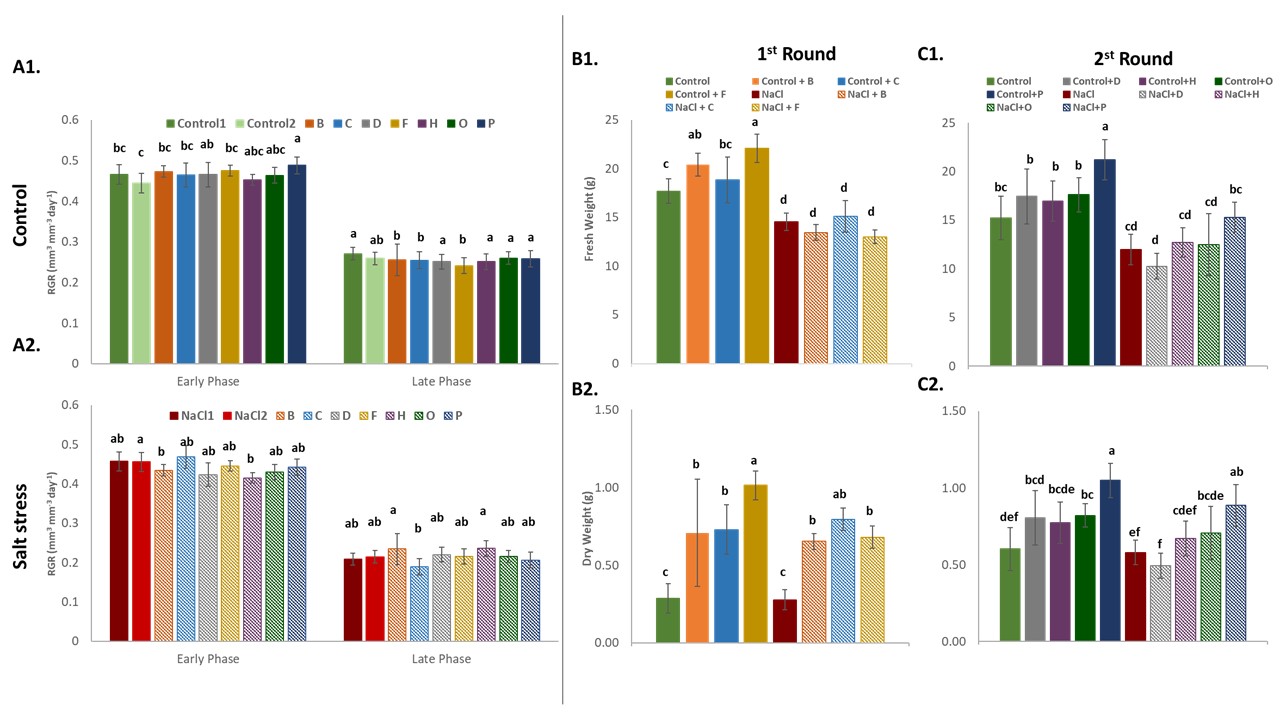

Supplement: Supplementary Figure 6 — Relative Growth Rate and final biomass of lettuce plants treated with PHs. Relative Growth Rate [RGR, (A1–A2)] of the different treatments over time, calculated for the early phase (from DoP 0 to DoP 12) and for the late Phase (from DoP 12 to DoP 21) in lettuce plants treated with 7 PHs grown under control or salt stress conditions. Total fresh (B1–B2) and dry (C1–C2) weight of the final aboveground biomass per variant. Values represent the average of the 8 biological replicates per variant. Error bars represent standard deviation. Different letters indicate significant differences according to the one-way ANOVA post hoc Tukey’s test (p < 0.05). [file Image_6.JPEG]

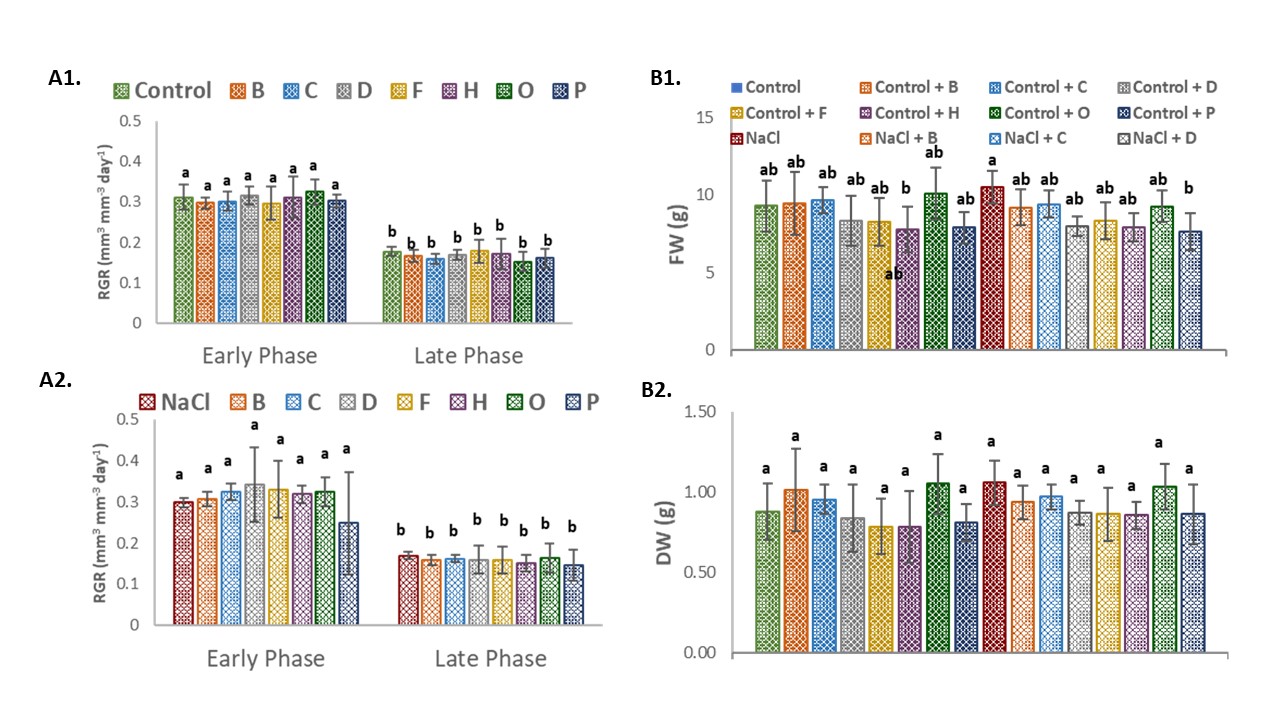

Supplement: Supplementary Figure 7 — Relative Growth Rate and final biomass of tomato plants treated with PHs. Relative Growth Rate [RGR, (A1–A2)] of the different treatments over time, calculated for the early phase (from DoP 0 to DoP 12) and for the late Phase (from DoP 12 to DoP 24) in tomato plants sprayed with 7 PHs, grown under control conditions or salt stress. Total fresh (B1–B2) and dry (C1–C2) weight of the final aboveground biomass per variant. Values represent the average of the 6 biological replicates per variant. Error bars represent standard deviation. Different letters indicate significant differences according to the one-way ANOVA post hoc Tukey’s test (p < 0.05). [file Image_7.JPEG]

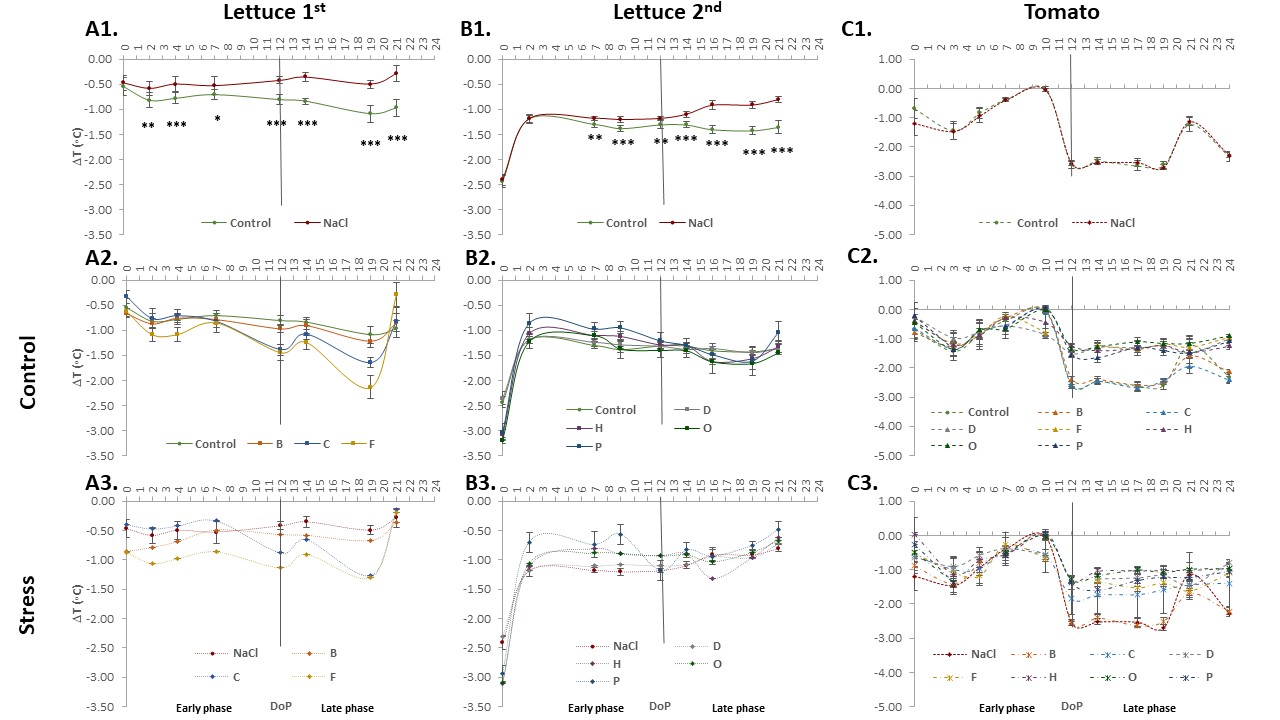

Supplement: Supplementary Figure 8 — Temperature of the leaves for lettuce and tomato plants under control and salt stress conditions. Canopy temperature depression measured on lettuce [(A1–A3, B1–B3), full and dotted lines] and tomato [(C1–C3), dashed and dotted + dashed lines] plants, untreated or treated with the biostimulant substances, grown under control or salt stress conditions. Values represent the average of the 8 biological replicates per treatment in lettuce and 6 biological replicates in tomato, error bars represent standard deviation. The significant differences between control and salt treatment are indicated with *, **, and *** for p-values below 0.05, 0.01, and 0.001, respectively. [file Image_8.JPEG]

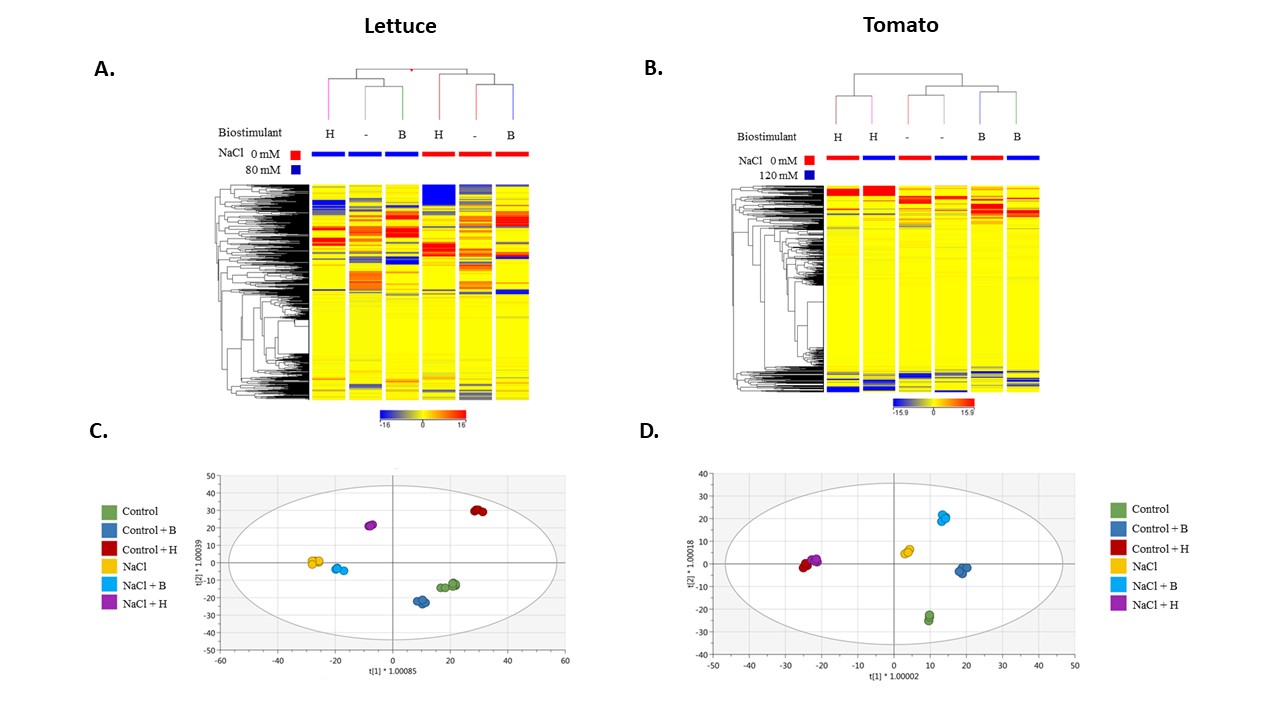

Supplement: Supplementary Figure 9 — Metabolomic analysis for the best (H) and worst (B) performing biostimulants. Unsupervised hierarchical cluster analysis carried out from UHPLC-ESI/QTOF-MS metabolomics analysis of lettuce (A) and tomato (B) plants treated with PH B or H, grown under control or salt stress conditions. The fold-change-based heat map was used to build hierarchical clusters (linkage rule: Ward, distance: Euclidean). Score plot of orthogonal projection to latent structures discriminant analysis (OPLS-DA) supervised modelling carried out on untargeted metabolomics profiles of lettuce (C) and tomato (D) plants after B and H application grown under control and salt stress (NaCl) condition. [file Image_9.JPEG]

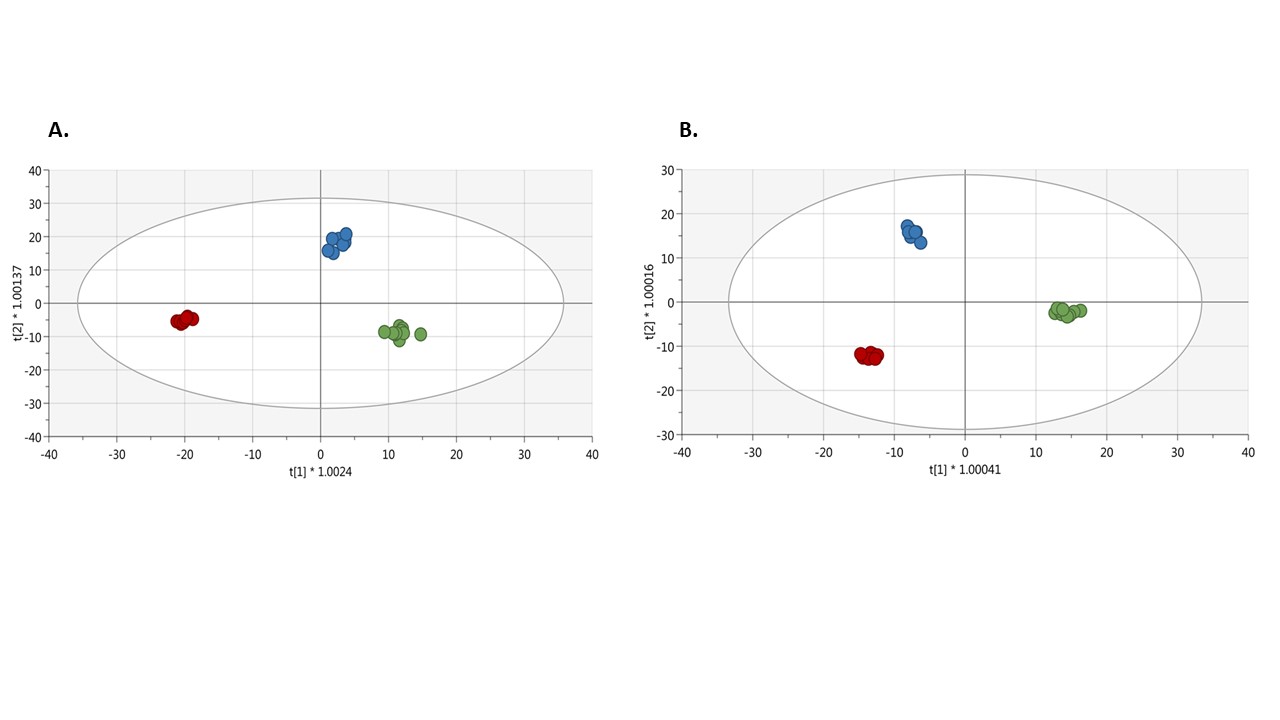

Supplement: Supplementary Figure 10 — Score plot of metabolomics profiles. Score plot orthogonal projection to latent structures discriminant analysis (OPLS-DA) supervised modelling carried out on untargeted metabolomics profiles of tomato and lettuce plants after B and H application grown under control (A) or salt stress (B) conditions. [file Image_10.JPEG]

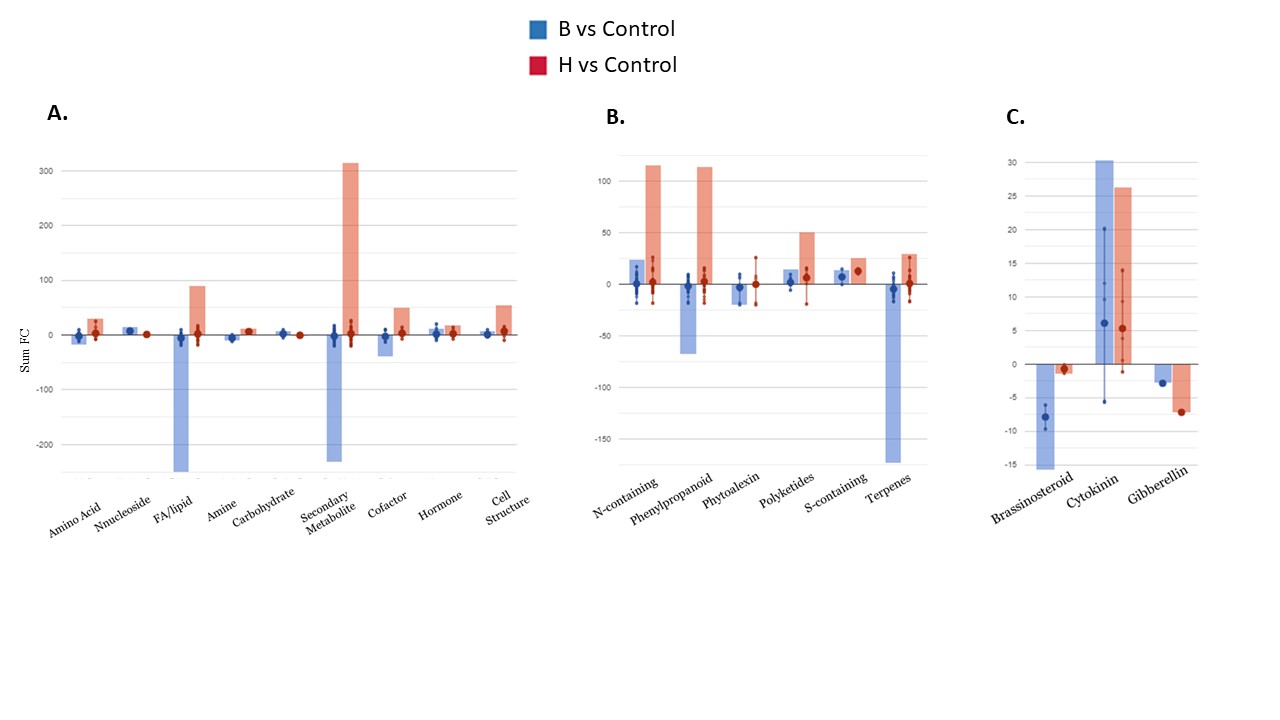

Supplement: Supplementary Figure 11 — Identified metabolites in control conditions. Metabolic processes (A), secondary metabolism (B), and hormone biosynthesis (C) were impaired by treatments in lettuce and tomato plants grown under control conditions. Differential metabolites (VIP score > 1.20) along with their fold-change (FC) values were elaborated using the Omic Viewer Dashboard of the PlantCyc pathway Tool software (www.pmn.plantcyc.com). The large dots represent the average (mean) of all log FC for metabolites, and the small dots represent the individual log FC for each metabolite. The x-axis represents each set of subcategories, while the y-axis corresponds to the cumulative log FC. FA/Lipid: fatty acids and lipids, Amine: amines and polyamines, Cofactor: cofactors, prosthetic groups, electron carriers, and vitamins, N-containing: Nitrogen-containing secondary metabolites, S-containing: Sulphur-containing secondary metabolites, Sugar Derives: sugar derivatives. [file Image_11.JPEG]

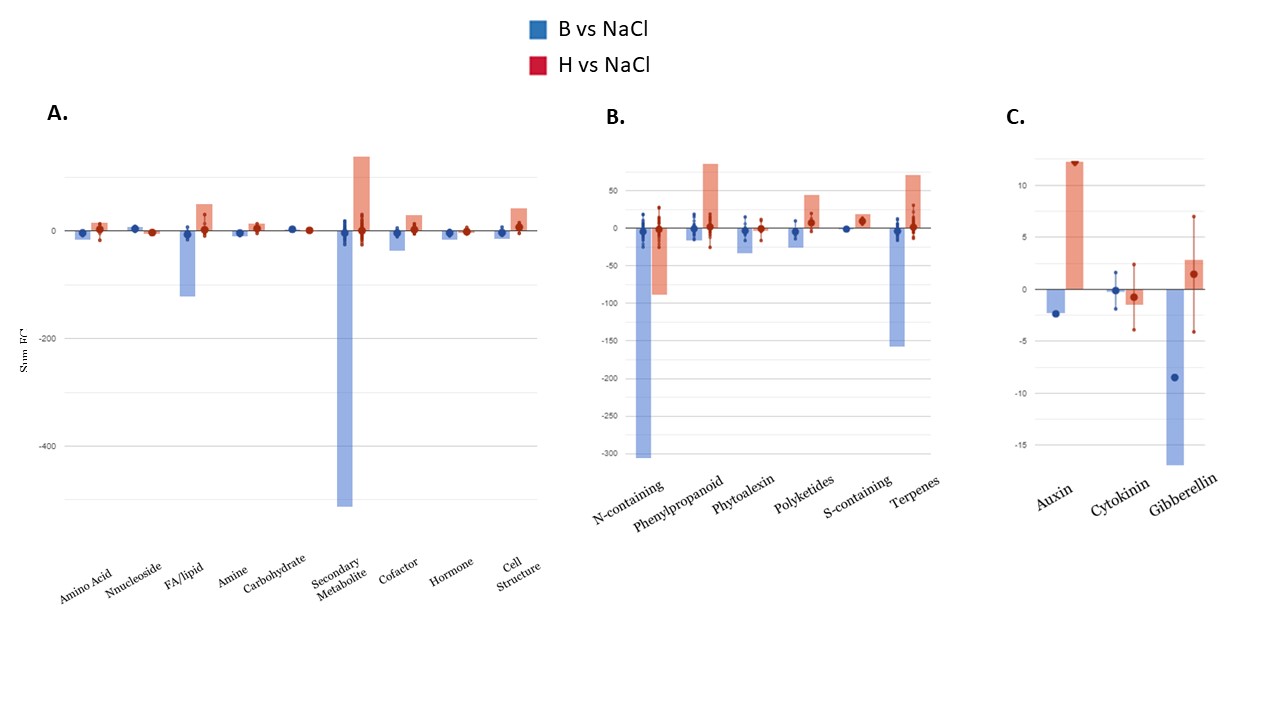

Supplement: Supplementary Figure 12 — Identified metabolites in plant under salt stress conditions. Metabolic processes (A), secondary metabolism (B), and hormone biosynthesis (C) were impaired by treatments in lettuce and tomato plants grown under salt stress conditions. Differential metabolites (VIP score > 1.20) along with their fold-change (FC) values were elaborated using the Omic Viewer Dashboard of the PlantCyc pathway Tool software (https://plantcyc.org/). The large dots represent the average (mean) of all log FC for metabolites, and the small dots represent the individual log FC for each metabolite. The x-axis represents each set of subcategories, while the y-axis corresponds to the cumulative log FC. FA/Lipid: fatty acids and lipids, Amine: amines and polyamines, Cofactor: cofactors, prosthetic groups, electron carriers, and vitamins, N-containing: Nitrogen-containing secondary metabolites, S-containing: Sulphur-containing secondary metabolites, and Sugar Derivs: sugar derivatives. [file Image_12.JPEG]

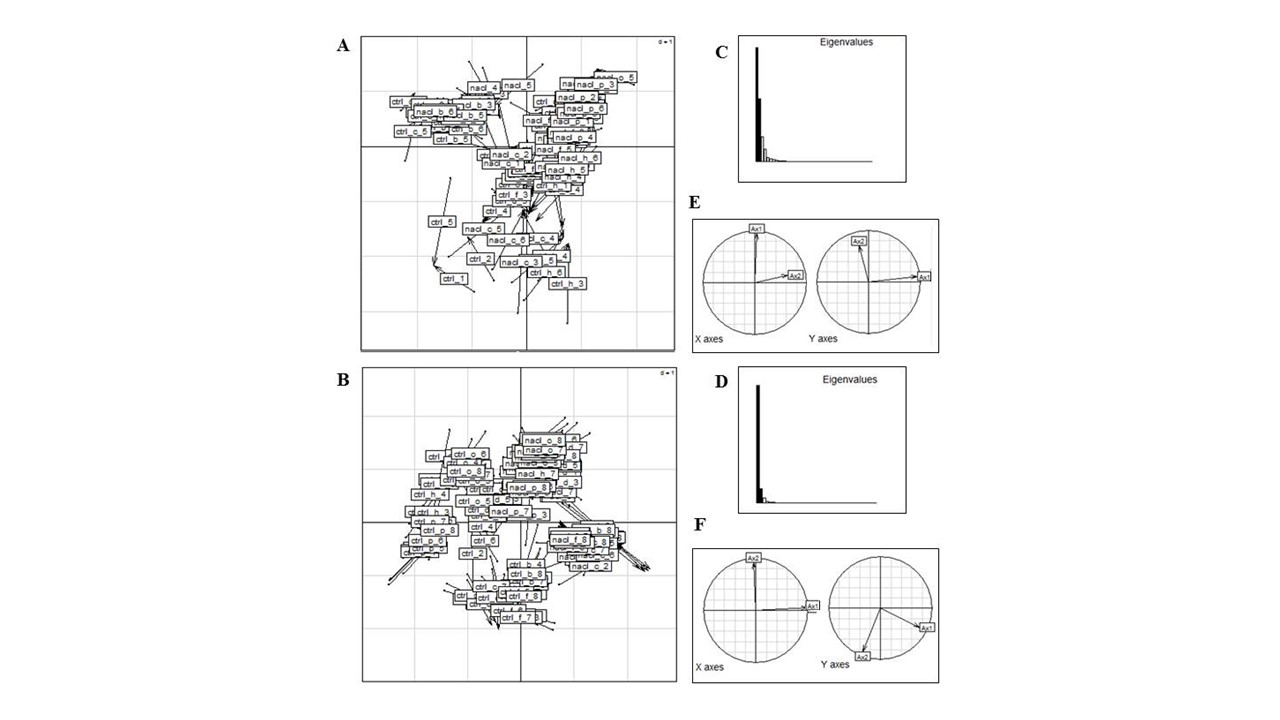

Supplement: Supplementary Figure 13 — Graphical output of co-Inertia analysis (CIA). Scatter plot of tomato (A) and lettuce (B) samples. Each sample is represented by an arrow whose length is proportional to the divergence between the phenomic and the metabolomic datasets. Eigenvalues of the co-inertia analysis for tomato (C) and lettuce (D). Correlation circles (E,F) showing the projections of the PCA axes (from the phenomic datasets) onto the axes of the co-inertia analysis (x axes) and projections of the PCA axes (from the metabolomic datasets) onto the axes of the co-inertia analysis (y axes). These four circles represent a view of the rotations needed to associate the two datasets for tomato (E) and the two datasets for lettuce (F). [file Image_13.JPEG]
